# BREC: an R package/Shiny app for automatically identifying heterochromatin boundaries and estimating local recombination rates along chromosomes

**DOI:** 10.1186/s12859-021-04233-1

**Published:** 2021-08-06

**Authors:** Yasmine Mansour, Annie Chateau, Anna-Sophie Fiston-Lavier

**Affiliations:** 1 Genomics Department, Institute of Evolution Science of Montpellier (ISEM), Montpellier, France; 2 Informatics Department, Laboratory of Computer Science, Robotics and Microelectronics of Montpellier (LIRMM), Montpellier, France; 3 Institut Universitaire de France (IUF), Paris, France

**Keywords:** Heterochromatin regions, Centromere position, Recombination rate, Non-genome-specific, Data quality control, Graphical user interface

## Abstract

**Background:**

Meiotic recombination is a vital biological process playing an essential role in genome's structural and functional dynamics. Genomes exhibit highly various recombination profiles along chromosomes associated with several chromatin states. However, eu-heterochromatin boundaries are not available nor easily provided for non-model organisms, especially for newly sequenced ones. Hence, we miss accurate local recombination rates necessary to address evolutionary questions.

**Results:**

Here, we propose an automated computational tool, based on the Marey maps method, allowing to identify heterochromatin boundaries along chromosomes and estimating local recombination rates. Our method, called **BREC** (heterochromatin **B**oundaries and **REC**ombination rate estimates) is non-genome-specific, running even on non-model genomes as long as genetic and physical maps are available. BREC is based on pure statistics and is data-driven, implying that good input data quality remains a strong requirement. Therefore, a data pre-processing module (data quality control and cleaning) is provided. Experiments show that BREC handles different markers' density and distribution issues.

**Conclusions:**

BREC's heterochromatin boundaries have been validated with cytological equivalents experimentally generated on the fruit fly *Drosophila melanogaster* genome, for which BREC returns congruent corresponding values. Also, BREC's recombination rates have been compared with previously reported estimates. Based on the promising results, we believe our tool has the potential to help bring data science into the service of genome biology and evolution. We introduce BREC within an R-package and a Shiny web-based user-friendly application yielding a fast, easy-to-use, and broadly accessible resource. The BREC R-package is available at the GitHub repository https://github.com/GenomeStructureOrganization.

**Supplementary Information:**

The online version contains supplementary material available at 10.1186/s12859-021-04233-1.

## Background

Meiotic recombination is a vital biological process that plays an essential role in investigating genome-wide structural and functional dynamics. Recombination events are observed in almost all eukaryotic genomes. Crossover, a one-point recombination event, is the exchange of DNA fragments between sister chromatids during meiosis. Recombination is a fundamental process that ensures genotypic and phenotypic diversity. Thereby, it is strongly related to various genomic features such as gene density, repetitive DNA, and DNA methylation [1–3].

Recombination rate varies not only between species but also within species and along chromosomes. Different heterochromatin regions exhibit different profiles of recombination events. Therefore, in order to understand how and why the recombination rate varies, it is vital to break down the chromosome structure into smaller blocks where several genomic features, besides recombination rate, are also known to exhibit different profiles. Chromatin boundaries allow to distinguish between two primary states of chromatin that can be defined as euchromatin, which is lightly compact with a high gene density, and on the contrary, heterochromatin, which is highly compact with a paucity in genes. The heterochromatin is represented in different chromosome regions: the centromere and the telomeres. Euchromatin and heterochromatin regions exhibit different behaviors in terms of genomic features and dynamics related to their biologic function, such as the cell division process that ensures the organism viability. Consequently, easily distinguishing chromatin states is necessary for conducting further studies in various research fields and to be able to address questions related to cellular processes such as meiosis, gene expression, epigenetics, DNA methylation, natural selection and evolution, genome architecture and organization, among others [4–6]. In particular, the profound understanding of centromeres, their complete and precise structure, organization, and evolution is currently a hot research area. These repeat-rich heterochromatin regions are currently still either poorly or not assembled at all across eukaryote genomes. Despite the enormous advances offered by the Next Generation Sequencing (NGS) technologies, centromeres are still considered enigmas, mostly because they prevent genome assembly algorithms from reaching their optimal performance to achieve more complete whole genome sequences [7]. Besides, the highly diverse mechanisms of heterochromatin positioning [8] and repositioning [9] remain a complicated obstacle in the face of fully understanding genome organization. Thus, generating high resolution genetic, physical, and recombination maps and locating heterochromatin regions is increasingly attractive to the community across an extensive range of taxa [10–16].

Numerous methods for estimating recombination rates exist. Genomic inference methods, covering population-based, pedigree-based and gamete-based approaches, have been included in the latest review by [17]. Among the listed methods, population genetic-based methods [18] provide accurate fine-scale estimates. Nevertheless, these methods are costly, time-consuming, require substantial expertise, and most of all, do not apply to all kinds of organisms. Moreover, the sperm-typing method [19], which is also extremely accurate, providing high-density recombination maps, is male-specific and is applicable only on limited genome regions. On the other hand, a purely statistical approach, the Marey Maps [20], could avoid some of the above issues based on other available genomic data: the genetic and physical distances of genomic markers.

The Marey maps approach consists of correlating the physical map with the genetic map representing respectively physical and genetic distances for a set of genetic markers on the same chromosome. Despite the efficiency of this approach and mostly the availability of physical and genetic maps, generating recombination maps rapidly and for any organism is still challenging. Hence, the increasing need for an automatic, portable, and easy-to-use solution.

Some Marey map-based tools already exist, two of which are primarily used. The MareyMap Online [21, 22] applies to multiple species, yet, it does not allow an accurate estimate of recombination rates on specific regions like the chromosome extremities. Second, the *Drosophila melanogaster* Recombination Rate Calculator (RRC) [23] solves the previous issue by adjusting recombination rate estimates on such chromosome regions, but as indicated by its name, it is *D. melanogaster*-specific. With the emerging NGS technologies, accessing whole chromosome sequences has become possible on a wide range of species. Therefore, we may expect an exponential increase in the markers number, requiring more adapted tools to handle such new scopes of data efficiently.

**Fig. 1:**
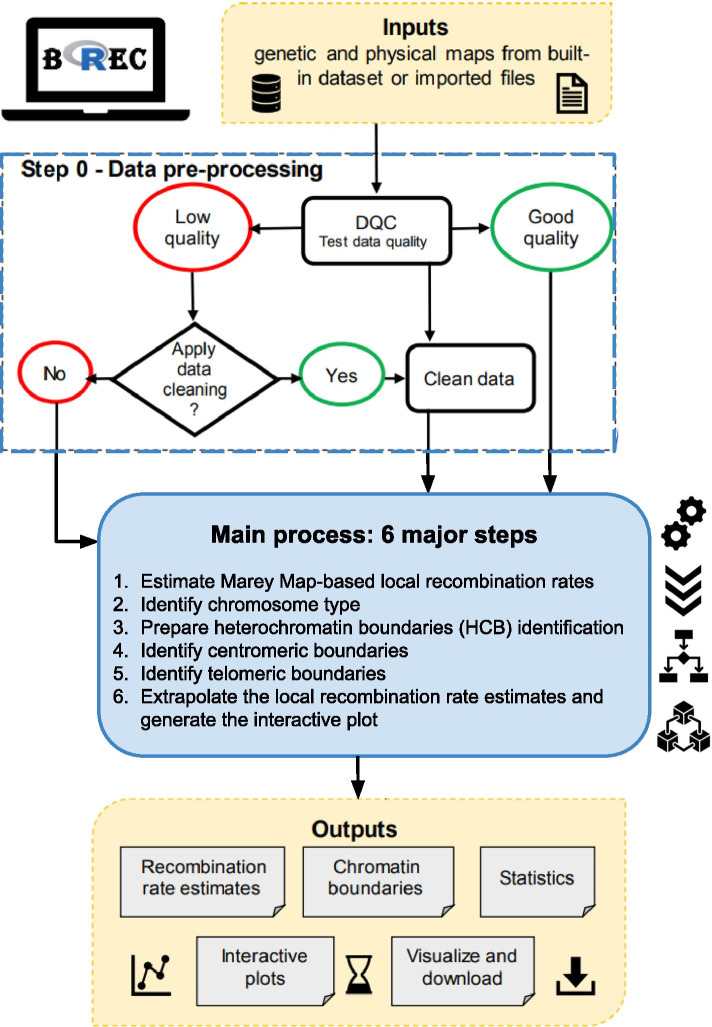
BREC workflow. This figure provides an overview of the tool design explaining how the different modules are linked together and how BREC functionalities are implemented. The top-to-bottom diagram starts with the required input data, how they are pre-processed (Step 0) and exploited (Main process: 6 major steps), then, what outputs are expected to be returned and in which format. A more detailed version is included in the Additional file 16, where a zoom-in on the main process is clarified for each of the six steps

Here, we propose a new Marey map-based method as an automated computational solution that aims to, firstly, identify heterochromatin boundaries (HCB) along chromosomes, secondly, estimate local recombination rates, and lastly, adjust recombination rates on chromosome along the chromosomal regions marked by the identified boundaries. Our proposed method, called **BREC** (heterochromatin **B**oundaries and **REC**ombination rate estimates), is provided within an R-package and a Shiny web-based graphical user interface. BREC takes as input the same genomic data, genetic and physical distances, as in previous tools. It follows a workflow (see Fig. 1) that, first, tests the data quality and offers a cleaning option, then estimates local recombination rates and identify HCB. Finally, BREC re-adjusts recombination rate estimates along heterochromatin regions, the centromere and telomere(s), in order to keep the estimates as authentic as possible to the biological process [24]. Identifying the boundaries delimiting euchromatin and heterochromatin allows investigating recombination rate variations along the whole genome, helping to compare recombination patterns within and between species. Furthermore, such functionality is fundamental for identifying the position of the centromeric and telomeric regions. Indeed, the position of the centromere along the chromosome has an influence on the chromatin environment, and recent studies are interested in investigating how genome architecture may change with centromere organization [7].

Our results have been validated with cytological equivalents, experimentally generated on the fruit fly *D. melanogaster* genome [4, 25, 26]. Moreover, since BREC is non-genome-specific, it could efficiently be run on other model as well as non-model organisms for which both genetic and physical maps are available. Even though it is still an ongoing study, BREC has also been tested with different species, and the results are reported.

This paper is organized as follows: the set of our results, based on both simulated and real data, are reported in "Results" section. They are then discussed in "Discussion" section. Concluding remarks with some perspectives are outlined in "Conclusions" section. The full set of BREC modules, detailed within a step-by-step workflow, as well as further details on the data involved, and how the methods were calibrated and validated, are presented in "Methods" section. Additional files: 1, 3, 4, 5, 7, 8, 9, 10, 11, 12, 13, 14, 16, 18, 19 consist of Figures S1–S15, and Additional files: 2, 6, 15, 17, 20, 21 include Tables S1-S6).

**Fig. 2:**
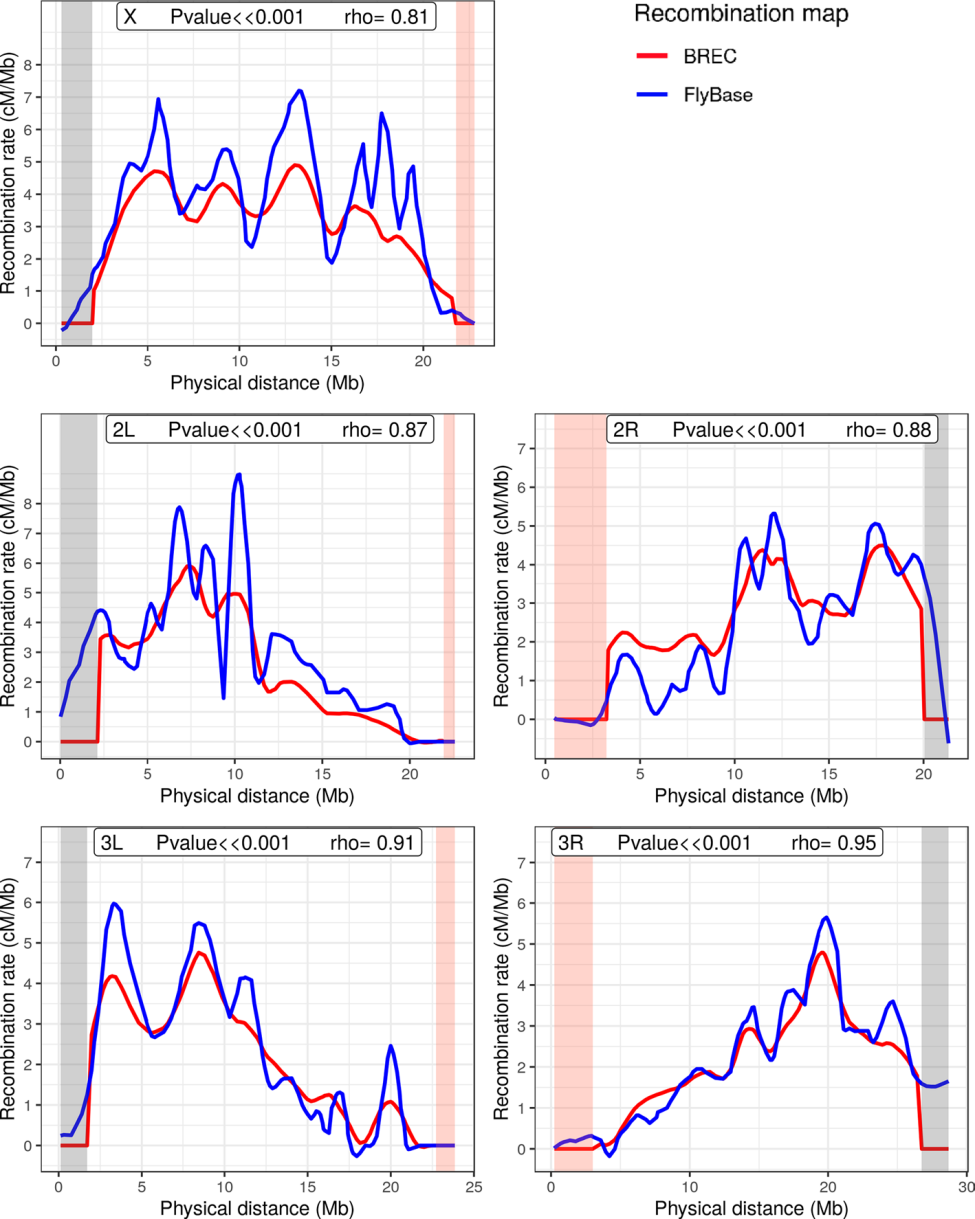
Comparison of BREC versus FlyBase recombination rate recombination rates along the five chromosomal arms (X, 2L, 2R, 3L, 3R) of D. melanogaster Release 5. Both recombination maps are obtained using the same regression model: Loess with span 15%. The HCB defined by BREC are represented in red and the reference data are in blue. Heterochromatin regions identified by BREC are highlighted in yellow

**Fig. 3:**
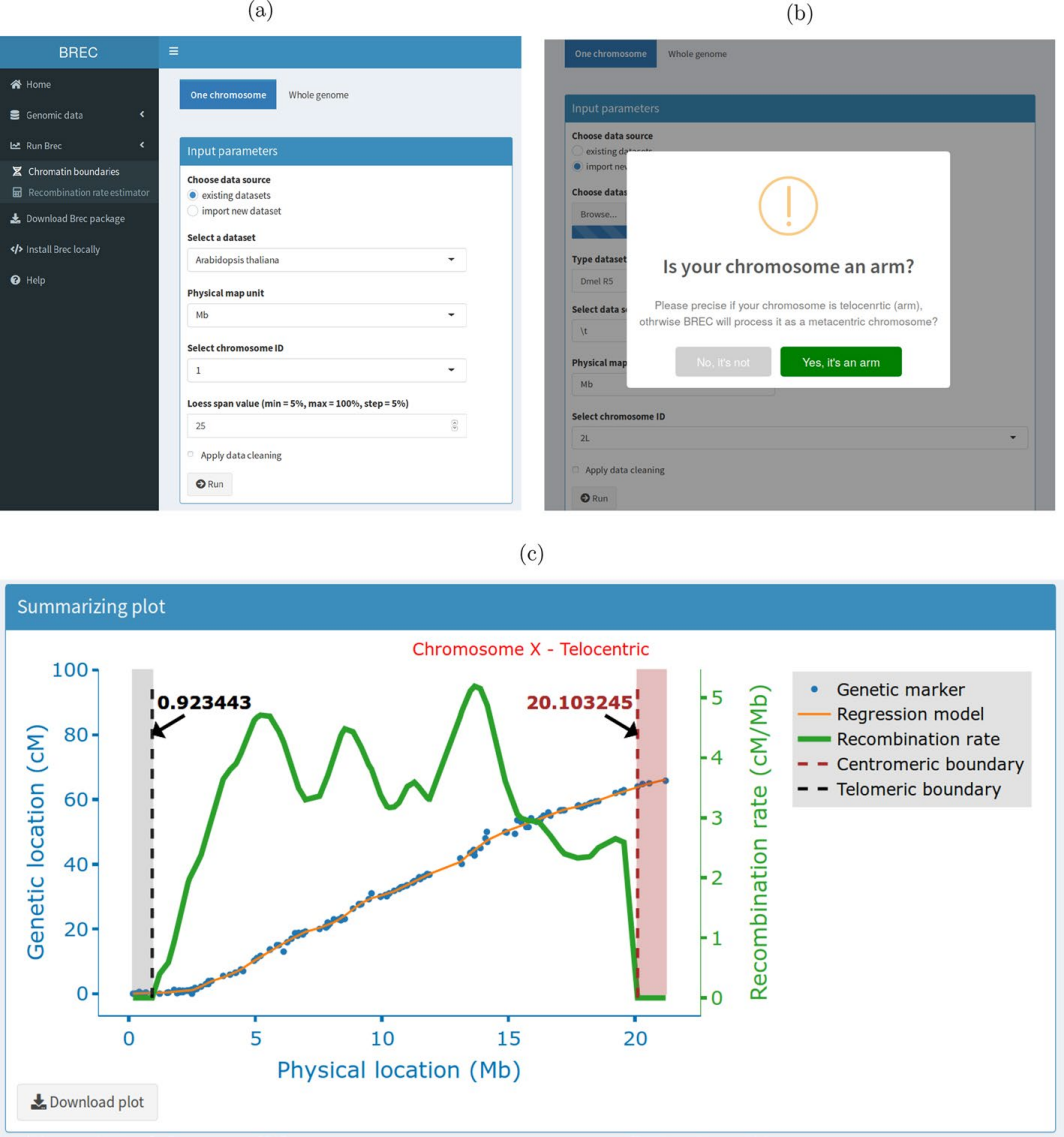
Screenshots of BREC web application - Run BREC web page **a** and **b** show the inputs interface. **c** It shows the output of running BREC on the specified inputs, represented with an interactive web-based plot as a result

## Results

In this section, we present the results obtained through the following validation process. First, we automatically re-identified HCB with an approximate resolution to the reference equivalents. Second, we tested the robustness of BREC methods according to input data quality, using the well-studied *D. melanogaster* genome data, for which recombination rate and HCB have already been accurately provided [4, 23, 25, 27] (Additional file 1). Besides, we extended the robustness test to a completely different genome, the domesticated tomato *S. lycopersicum* [28] to better interpret the study results. Even if the Loess span value does not impact the HCB identification, but only the resulting recombination rate estimates, the span values used in this study are: 15% for *D. melanogaster* (for comparison purpose) and 25% for the rest of the experiments. Our analysis shows that BREC is applicable to data from various organisms, as long as the data quality is good enough. BREC is data-driven, thus, the outputs strongly depend on the markers density, distribution, and chromosome type identified (automatically, or with the user's a priori knowledge).

### Approximate, yet congruent HCB

#### Fruit fly genome *D.melanogaster*

Our approach for identifying HCB has been primarily validated with cytological data experimentally generated on the *D. melanogaster* Release 5 genome [4, 25, 26, 29]. For all five chromosomal arms (X, 2L, 2R, 3L, 3R). This genome presents a mean density of 5.39 markers/Mb and a mean physical map length of 22.92Mb. We obtained congruent HCB with a good overlap and shift, distance between the physical position of the reference and BREC, from 20Kb to 4.58Mb (see "Data and implementation" section). We did not observe a difference in terms of mean shift for the telomeric and centromeric BREC identification ($\chi ^2 = 0.10$, df = 1, $p-value = 0.75$)(See Table 1 and Additional file 2). We observe a lower resolution for the chromosomal arms 3L and 3R (see Additional file 3). This suggests that those two chromosomal arms' data might not present as good quality as the rest of the genome. Interestingly, the local markers density for these two chromosomal arms shows a high variation, unlike the other chromosomal arms. For instance, the 2L for which BREC returns accurate results, shows a lower variation (see Additional file 4). Without these two arms, the max shift for both centromeric and telomeric BREC boundaries is smaller than 1.54Mb, with a mean shift decreasing from 1.43 to 0.71 Mb.

**Table 1:** BREC HCB compared to reference boundaries from the reference genome of ***D. melanogaster***

Chromosomal arm	Centromeric (Mb)			Telomeric (Mb)		
	Boundaries		Shift	Boundaries		Shift
	Reference	BREC		Reference	BREC	
X	20.67	20.10	0.56	2.46	0.92	1.54
2L	19.95	20.33	0.38	0.70	0.68	0.02
2R	6.09	5.01	1.08	20.02	20.71	0.69
3L	18.41	20.30	1.90	0.36	2.26	1.91*
3R	8.35	3.77	4.58*	27.25	25.64	1.61
Min. shift	0.38			0.02		
Max. shift	4.58			1.91		
Mean shift	1.70			1.15		
Median shift	1.08			1.54		

The shift is the absolute value of the distance between the BREC and the reference physical heterochromatin boundary. The first five rows represent all chromosomal arms. Grouped columns present reference, BREC and shift values for the centromeric boundaries (Columns 2–4), and for the telomeric boundaries (Columns 4–6). Here the boundary values correspond to the internal HCB. The external boundaries are represented by the physical positions of the first and the last markers of the chromosomes. All values are expressed in Megabase (Mb). The asterisk indicates the largest shift value reported on centromeric and telomeric boundaries separately (see corresponding Additional file 3). The last four rows represent general statistics on the shift value. From top to bottom, they are minimum, maximum, mean, and median respectively. See details on the shift metrics in "Validation metrics" section

This first analysis suggests that BREC methods return accurate results on this genome. However, the boundaries identification process appears very sensitive to the markers' local density and distribution along a chromosome (see Additional file 3). Therefore, we conducted further experiments on a different dataset, the tomato genome (see Additional file  5).

#### Tomato genome *S. lycopersicum*

Results of experimenting BREC behaviour on all 12 chromosomes of *S. lycopersicum* genome [28] are shown as values in Additional file 6 and as plots in Additional file 7. This genome presents a mean density of 2.64 markers/Mb and a mean physical map length of 62.71Mb. We observe a variation in the shift value representing the difference on the physical map between reference HCB and their equivalents returned by BREC. Unlike the *D. melanogaster* genome, which is of a smaller size, with five telocentric chromosomes (chromosomal arms) and a strongly different markers distribution, the tomato genome exhibits a completely different study case. It is a plant genome, with approximately 8-fold bigger genome size. It is organized as twelve atelocentric chromosomes of a mean size of  60Mb, except for chromosomes 2 and 6, which are more likely to be rather considered telocentric based on their markers distribution. Also, we observe a long plateau of markers along the centromeric region with lower density than the rest of the chromosomes. Something which highly differs from *D. melanogaster* data. We believe all these differences between both genomes give a good validation and evaluation for BREC behavior towards various data quality scenarios. Furthermore, since BREC is a data-driven tool, these experiments help analyze data-related limitations that BREC could face while resolving differently. From another point of view, BREC results on the tomato genome highlight the fact that markers distribution along heterochromatin regions, in particular, strongly impacts the identification of eu-heterochromatin boundaries, even when the density is of 2 markers/Mb or more.

### Consistency despite the low data quality

We aim in this part to study to what extent BREC results are depending on the data quality.

#### BREC handles low markers density

We started by assessing the markers' density on the BREC estimates. We generated simulated datasets with decreasing fractions of markers for each chromosomal arm (from 100% to 30%). For that, we randomly selected a fraction of markers, 30 times, and computed the mean shift between BREC and the reference telomeric and centromeric boundaries. We have noted that BREC's resolution decreases drastically with the fraction and therefore with the marker density (see Additional file 8). However, BREC results appeared stable until 70% of the data for all the chromosomal arms, more specifically for the telomeric boundary detection. Only for the centromeric boundary of the chromosomal arm 3R, we observed the opposite pattern: BREC returns more accurate telomeric boundary estimates when the markers' number decreases. This supports the low quality of the data around the 3R centromere.

This simulation process allowed to set a minimum density threshold representing the minimum value for data density in order to guarantee accurate results for BREC estimates at 5 markers/Mb (fraction of around 70% of the data) on average in *D. melanogaster*. This analysis also supports the fact that because the markers' density alone can not explain the BREC resolution, BREC may also be sensitive to the marker distribution.

Additional file 4 clearly shows that markers' density varies within and between the five chromosomal arms with a mean of 4 to 8 markers/Mb. The variance is induced by the extreme values of local density, such as 0 or 24 markers/Mb on the chromosomal arm X. Still, the overall density is around 5 markers/Mb for the whole genome.

#### BREC handles heterogeneous distribution

Along chromosomes, genetic markers are not homogeneously distributed. Therefore, to assess the impact of the distribution of markers on BREC results, we designed different *data scenarios* regarding a reference data distribution (see "Simulated data for quality control testing" section). We choose as reference the chromosomal arms 2L and 2R of *D. melanogaster* as we have obtained the most accurate results with their data. After the concatenation of the two arms, we ended up with a metacentric simulated chromosome as a starting simulation scenario (total physical length of 44Mb). While this length was kept unchanged, markers local density and distribution were modified (see "Simulated data for quality control testing" section and Additional file 9).

One particular yet typical case is the centromeric gap. Throughout our analysis, we consider that a chromosome presents a centromeric gap if its data exhibit a lack of genetic markers on a relatively large region on the physical map. Centromeric regions usually are less accessible to sequence due to their highly compact chromatin state. Consequently, these regions are also hard to assemble, and that is why many genomes have chromosomes presenting a centromeric gap. It is essential to know that a centromeric gap is not always precisely located in the middle of a chromosome. Instead, its physical location depends on the chromosome type (see more details in Additional file 10).

We also assess the veracity of BREC on datasets with variable distributions using simulated data with and without a centromeric gap (see Additional file 9).

For all six simulation datasets, BREC results overlap the reference boundaries. Thus BREC correctly handles the presence of a centromeric gap (see Additional file 9: (a)(c)(e)). BREC remains robust to a non-uniform distribution of markers, under the condition that regions flanking the boundaries are greater than 2 markers/Mb (see Additional file 11). In the case of a non-uniform distribution, BREC resolution is higher when the local density is stronger around heterochromatin regions (see Additional file 9: (c)(d)(e)(f)). This suggests that low density on euchromatin regions far from the boundaries is not especially a problem either.

### Accurate local recombination rate estimates

After identifying the HCB, BREC provides optimized local estimates of recombination rate along the chromosome by taking into account the absence of recombination in heterochromatin regions. Recombination rates are reset to zero across the centromeric and telomeric regions regardless of the regression model. To closely compare the third degree polynomial with Loess, using different span values, we experimented with this aspect on *D. melanogaster* chromosomal arms and reported the results in Additional file 12.

To assess the veracity of the recombination rates along the whole genome, we compared BREC results with previous recombination rate estimates (see Fig. 2; [4, 25]). BREC recombination rate estimates are significantly strongly correlated with reference data (Spearman's: $P\ll 0.001$) while the reference estimates fail in telomeric regions.

### BREC is non-genome-specific

NGS, High Throughput Sequencing (HTS) technologies, and numerous further computational advances are increasingly providing genetic and physical maps with more and more accessible markers along the centromeric regions. Such progress in the availability of data of poorly accessible genomic regions is a huge opportunity to shift our knowledge of heterochromatin DNA sequences and their dynamics, as in the case of Transposable Elements (TEs), for example. Therefore, BREC is not identifying centromeric gaps as centromeric regions as it might seem. Instead, it is targeting centromeric as well as telomeric boundaries identification regardless of the presence or absence of markers neither of their density or distribution variations across such complicated genomic regions (see Additional file 13). Given that BREC is non-genome-specific, applying HCB identification on various genomes has allowed to widen the experimental design and to test more thoroughly how BREC responds to different *data scenarios*. Despite the several challenges due to data quality issues and following a data-driven approach, BREC is a non-genome-specific tool that aims to help to tackle biological questions.

### Easy, fast and accessible tool via an R-package and a Shiny app

BREC is an R-package entirely developed with the R programming language. The current version of the package and documentation are available on the GitHub repository: https://github.com/GenomeStructureOrganization.

In addition to the interactive visual results provided by BREC, the package comes with a web-based Graphical User Interface (GUI) build using the shiny and shinydashboard libraries. The intuitive GUI makes it a lot easier to use BREC without struggling with the command line (see screenshots in Fig. 3d and Additional file 14).

As for the speed aspect, BREC is quite fast when executing the main functions. We reported the running time for *D. melanogaster R5* and *S. lycopersicum* in Additional files 2 and 15, respectively (plotting excluded). Nevertheless, when running BREC via the Shiny application, and due to the interactive plots displayed, it takes longer because of the plotly rendering. Still, it depends on the size of the genetic and physical maps used, as well as the markers density, as slightly appears in the same tables. The results presented from other species (see Additional file 13) highlight better this dependence.

## Discussion

The main two results of BREC are the eu-heterochromatin boundaries and the local recombination rate estimates (see Fig. 2 and Additional file  3).

The HCB algorithm, which identifies the location of centromeric and telomeric regions on the physical map, relies on the regression model obtained from the correlation between the physical distance and the genetic distance of each marker. Then, the goodness-of-fit measure, the R-squared, is used to obtain a curve upon which the transition between euchromatin and heterochromatin is detectable.

On the other hand, the recombination rate algorithm, which estimates local recombination rates, returns the first derivative of the previous regression model as the recombination rates, then resets the derivative values to zero along the heterochromatin regions identified (see Additional file 16).

We validated BREC methods with a reference dataset known to be of high quality: *D. melanogaster*. While two distinct approaches were respectively implemented for the detection of telomeric and centromeric regions, our results show a similar high resolution (see Table 1 and Additional file 3). Then we analysed BREC's robustness using simulations of a progressive data degradation (see Additional files 8 and 11). Even if BREC is sensitive to the markers' distribution and thus to the local markers' density, it can correctly handle a low global markers' density. For the *D. melanogaster* genome, a density of 5 markers/Mb seems to be sufficient to detect the HCB accurately.

We also validated BREC using the domesticated tomato *S. lycopersicum* dataset (see Additional files 6 and  7). At first glance, one might ask: why validating with this species when the results do not seem really congruent? In fact, we have decided to investigate this genome as it provides a more insightful understanding of the data-driven aspect of BREC and how data quality strongly impacts the heterochromatin identification algorithm. Variations in the local density of markers in this genome are particularly associated with the relatively large plateaued centromeric region representing more than 50% of the chromosome's length. Such *data scenario* is quite different from what we previously reported on the *D. melanogaster* chromosomal arms. This is partially the reason for which we chose this genome for testing BREC limits.

While analyzing the experiments more closely, we found that BREC processes some of the chromosomes as presenting a centromeric gap, while that is not actually the case. Thus, we forced the HCB algorithm to automatically apply the *with-no-centromeric-gap-algorithm*, then, we were inspired to implement this option into the GUI in order to give the users the ability to take advantage of their *a priori* knowledge and by consequence to use BREC more efficiently. Meanwhile, we are considering how to make BREC completely automated regarding this point for an updated version later on. Besides, the reference heterochromatin results we used for the BREC validation are rather an approximate than an exact indicator. The physical positions used as reference correspond to the first and last markers tagged as "heterochromatin" on the spreadsheet file published by the Tomato Genome Consortium authors in [28]. However, we hesitated before validating BREC results with these approximate reference values due to the redundant existence of markers tagged as "euchromatin" directly before or after these reference positions. Unfortunately, we were unable to validate telomeric regions since the reference values were not available. As a result, we are convinced that BREC is approximating well enough in the face of all the disrupting factors mentioned above.

On the other hand, this method's ambition is to escape species-dependence, which means it is conceived to apply to a various range of genomes. To test that, we also launched BREC on genomic data from different species (the house mouse's chromosome 4, roundworm's chromosome 3, and the chromosome 1 of zebrafish). Experiments on these whole genomes showed that BREC works as expected and identifies chromosome types in 95% of cases (see Additional file 13).

One can assume, with the exponential increase of genomic resources associated with the revolution of the sequencing technologies, that more fine-scale genetic maps will be available. Therefore, BREC has quite the potential to widen the horizon of deployment of data science in the service of genome biology and evolution. It will be crucial to develop a dedicated database to store all this data.

BREC package and design offer numerous advantageous functionalities (see Additional file 17) compared to similar existing tools [22, 23]. Thus, we believe our new computational solution will allow a large set of scientific questions, such as the ones raised by the authors of [5, 30], to be addressed more confidently, considering model as well as non-model organisms, and with various perspectives.

## Conclusions

We designed a user-friendly tool called BREC that analyses genomes on the chromosome scale, from the recombination point-of-view. BREC is a rapid and reliable method designed to determine euchromatin-heterochromatin boundaries on chromosomal arms or whole chromosomes (resp. telocentric or metacentric). BREC also uses its heterochromatin boundary results to improve the recombination rate estimates along the chromosomes.

Currently, the Shiny app is being deployed on the https://shinyapps.io server, in order to provide an install-free experience to the users. In addition, the "whole genome" version of BREC is a work in progress. It will allow to run BREC on all the chromosomes of a genome of interest at once. This version might also present the identified heterochromatin regions on chromosome ideograms. As short-term perspectives for this work, we may consider extending the robustness tests to additional datasets with high quality and mandatory information (*e.g.* boundaries identified with the cytological method, high quality maps). Retrieving such datasets seems to become less and less complicated. We may also improve the identification of boundaries with a more refined analysis around them, using an iterative multi-scale algorithm for instance. Finally, we will be happy to consider the users' feedback and improve our tool's ergonomy and usability. As mid-term perspectives, we underline that BREC could integrate other algorithms aiming to provide further analysis options such as the comparison of heterochromatin regions between closely related species. Also, we are aware that it would be interesting to compare BREC results with more existing methods. Thus, we plan to properly do so in the near future.

## Methods

### New approach: BREC

BREC is designed following the workflow represented in Fig. 1. To ensure that the broadest range of species could be analyzed by our tool, we designed a pipeline that adapts behavior with respect to input data. Each step of the workflow relies mostly on statistical analysis, adaptive algorithms, and decision proposals led by empirical observation.

The workflow starts with a pre-processing module (called "Step 0") aiming to prepare the data prior to the analysis. Then, it follows six main steps: (1) estimate Marey Map-based local recombination rates, (2) identify chromosome type, (3) prepare the HCB identification, (4) identify the centromeric boundaries, (5) identify the telomeric boundaries, and (6) extrapolate the local recombination rate map and generate an interactive plot containing all BREC outputs (see Fig. 1). Each step is detailed hereafter and summarised in Additional file 16.

#### Step 0 - Apply data pre-processing

Since we have noticed that BREC estimates are sensitive to the quality of input data, we propose a pre-processing step to assess data quality and suggest an optional data cleaning for outliers. As such, we could ensure proper functioning during further steps.

*Data quality control (DQC)* The quality of input data is tested regarding two criteria: (1) the density of markers and (2) the homogeneity of their distribution on the physical map along a given chromosome. First, the mean density, defined as the number of markers per physical map length, is computed. This value is compared with the minimum required threshold of 2 markers/Mb. Based on the displayed results, the user gets to decide if data cleaning is required or not. The threshold of 2 markers/Mb is selected based on a simulation process that allowed to test BREC results while decreasing markers density until the observed HCB estimates seemed to be no longer exploitable (see "Simulated data for quality control testing" section). Second, the distribution of input data is tested via a comparison with a simulated uniform distribution of identical markers density and physical map length. This comparison is applied using Pearson's $\chi^2$ test [31], which allows examining how close the observed distribution (input data) is to the expected one (simulated data).

*Data cleaning* The cleaning step aims to reduce the disruptive impact of noisy data, such as outliers, in order to provide a more accurate recombination rate and heterochromatin boundary results. If the input data fails to pass the Data Quality Control (DQC) test, the user has the option to apply or not a cleaning process. This process consists of identifying the extreme outliers and eliminating them upon the user's confirmation. Outliers are detected using the distribution statistics of the genetic map (see Additional file 18). More precisely, inter-marker distances (separating each two consecutive points) are computed along the genetic map. Using a boxplot, distribution statistics (quartiles, mean, median) are applied on these inter-marker distances to identify outliers, which are chosen as the 5% of the data points with a greater genetic distance than the maximum extreme value, and should be discarded. Thus, the cleaning targets markers for which the genetic distance is quite larger than most of the rest. After the first cleaning iteration, DQC is applied again to assess the new density and distribution. The user can also choose to bypass the cleaning step, but BREC's behavior is no longer guaranteed in such cases.

#### Step 1 - Estimate Marey Map-based local recombination rates

Once the data are cleaned, the recombination rate can be estimated based on the Marey map [20] approach by: (1) correlating genetic and physical maps, (2) generating two regression models -third degree polynomial and Loess- that better fits these data, (3) computing the prime derivative for both models which will represent preliminary recombination maps for the chromosome. The primary purpose of interpolation here is to provide local recombination rate estimates for any given physical position, instead of only the ones corresponding to available markers.

At this point, both recombination maps are used to identify the chromosome type as well as the approximate position of centromeric and telomeric regions. Nevertheless, as a final output, BREC will return only the Loess-based adjusted map for recombination rates since it provides finer local estimates than the polynomial-based map.

#### Step 2 - Identify chromosome type

BREC provides a function to identify the type of a given chromosome according to the position of its centromere. This function is based on the physical position of the smallest value of recombination rate estimates, which primarily indicates where the centromeric region is more likely to be located. Our experimentation allowed to come up with the following scheme (see Additional file 10). Two main types are identified: telocentric and atelocentric [32]. Atelocentric type could be either metacentric (centromere located approximately in the center with almost two equal arms) or not metacentric (centromere located between the center and one of the telomeres). The latter includes the two most known subtypes, submetacentric and acrocentric (recently considered types rather than subtypes). It is tricky for BREC to distinguish between submetacentric and acrocentric chromosomes correctly. Their centromeres' position varies slightly, and capturing this variation (based on the smallest value of recombination rate on both maps -polynomial and Loess-) could not be achieved yet. Therefore, we chose to provide this result only if the implemented process allowed to identify the subtype automatically. Otherwise, the user gets the statistics on the chromosome's data and is invited to decide according to further *a priori* knowledge. The two subtypes (metacentric and not metacentric) are distinguished following intuitive reasoning inspired by their definition found in the literature. First, BREC identifies whether the chromosome is an arm (telocentric) or not (atelocentric). Then, it tests if the physical position of the smallest value of the estimated recombination rate is located between 40% to 60% interval. In this case, the subtype is displayed as *metacentric*. Otherwise, it is displayed as *not metacentric*. The recombination rate is estimated using the Loess model ("LOcal regrESSion") [33, 34].

#### Step 3 - Prepare the HCB identification

The HCB identification is a purely statistical approach relying on the coefficient of determination $R^2$, which measures how good the generated regression model fits the input data [35]. We chose this approach because the Marey map usually exhibits a lower quality of markers (density and distribution) on the heterochromatin regions. Thus, we aim to capture this transition from high to low quality regions (or vice versa) as it reflects the transition from euchromatin to heterochromatin regions (or vice versa). The coefficient $R^2$ is defined as the cumulative sum of squares of differences between the interpolation and observed data. $R^2$ values are accumulated along the chromosome. In order to eliminate the biased effect of accumulation, $R^2$ is computed twice: $R^2-forward$ starts the accumulation from the beginning of the chromosome to provide the left centromeric and left telomeric boundaries. In contrast, $R^2-backwards$ starts from the end of the chromosome, providing the right centromeric and right telomeric boundaries. These $R^2$ values were calculated using the rsq package in R. To compute $R^2$ cumulative vectors, rsq function is applied on the polynomial regression model. In fact, there is no such function for non-linear regression models like the Loess because, in such models, high $R^2$ does not always indicate a good fit. A sliding window is defined and applied on the $R^2$ vectors to precisely analyze their variations (see details in the next step). In the case of a telocentric chromosome, the position of the centromere is then deduced as the left or the right side of the arm, while in the case of an atelocentric chromosome, the existence of a centromeric gap is investigated.

#### Step 4 - Identify centromeric boundaries

Since the centromeric region is known to present reduced recombination rates, the starting point for detecting its boundaries is the physical position corresponding to the smallest polynomial-based recombination rate value. A sliding window is then applied to expand the starting point into a region based on $R^2$ variations in two opposite directions. The sliding window's size is automatically computed for each chromosome as the largest value of ranges between each two consecutive positions on the physical map (indicated as *i* and $i+1$ in Eq. 1). After making sure the sliding window includes at least two data points, the mean of local growth rates inside the current window is computed and tested compared to zero. If it is positive (resp. negative) on the forward (resp. backward) $R^2$ curve, the value corresponding to the window's ending edge is returned as the left (resp. right) boundary. Else, the window moves by a step value equal to its size.

1 $$\begin{aligned} sliding\_window\_size(chromosome)= max\{|physPos_{i+1} - physPos_i| : 1 \le i \le n-1\} \end{aligned}$$

There are some cases where chromosome data present a centromeric gap. Such a lack of data produces biased centromeric boundaries. To overcome this issue, chromosomes with a centromeric gap are handled with a slightly different approach. After comparing the mean of local growth rates regarding to zero, accumulated slopes of all data points within the sliding window are computed, adding one more point at a time. If the mean of accumulated slopes keeps the same variation direction as the mean of growth rates, the centromeric boundary is set as the window's ending edge. Else, the window slides by the same step value as before (equal to its size). The difference between the two chromosome types is that only one sliding window is used for the telocentric case, its starting point is the centromeric side, and it moves away from it. As for the atelocentric case, two sliding windows are used (one on each $R^2$ curve), their starting point is the same, and they move in opposite directions to expand the centromere into a region.

#### Step 5 - Identify telomeric boundaries

Since telomeres are considered heterochromatin regions as well, they also tend to exhibit low fitness between the regression model and the data points. More specifically, the accumulated $R^2$ curve tends to present a significant depletion around telomeres. Therefore, a telomeric boundary is defined here as the physical position of the most significant depletion corresponding to the smallest value of the $R^2$ curve. As such, in the telocentric case, only one $R^2$ curve is used. It gives one boundary of the telomeric region (the other boundary is defined by the beginning of the left telomere or the end of the right telomere). Whilst in the atelocentric case, where the are two telomeres, the depletion on $R^2-forward$ detects the end of the left telomeric region, and the depletion on $R^2-backwards$ detects the beginning of the right telomeric region. The other two boundaries (the beginning of the left telomere and the end of the right telomere) are defined to be, respectively, the same values of the two markers with the smallest and the largest physical position available within the input data of the chromosome of interest.

#### Step 6 - Extrapolate the local recombination rate estimates and generate interactive plot

The extrapolation of recombination rate estimates at the identified centromeric and telomeric regions automatically performs an adjustment by resetting the initial biased values to zero along these heterochromatin ranges. Finally, all of the above BREC outputs are combined to generate one interactive plot to display for visualization and download (see details in "Easy, fast and accessible tool via an R-package and a Shiny app" section).

It is important to emphasize that throughout the whole main process module, only Step 1 " Estimating Marey map-based local recombination rates " comes from previous methods ([20, 21]). Otherwise, each of the steps 2-6 are fully developed (designed and implemented) within BREC and represent a new contribution, in addition to step zero " Data pre-processing ", as mentioned above.

### Data and implementation

#### Validation data

The only input dataset to provide for BREC is genetic and physical maps for one or several chromosomes. A simple CSV file with at least two columns for both maps is valid. If the dataset is for more than one chromosome or the whole genome, a third column, with the chromosome identifier, is required.

Our results have been validated using Release 5 of the fruit fly *D. melanogaster* [36, 37] genome as well as the domesticated tomato *Solanum lycopersicum* genome (version SL3.0).

We also tested BREC using other datasets of different species: house mouse (*Mus musculus castaneus*, MGI) chromosome 4 [38], roundworm (*Caenorhabditis elegans*, ws170) chromosome 3 [39], zebrafish (*Danio rerio*, Zv6) chromosome 1 [40], respectively (see Additional file 13), as samples from the multi-genome dataset included within BREC (see further details on the full built-in dataset in "Description of main components of the Shiny app" section).

*Fruit fly genome* *D.melanogaster* Physical and genetic maps are available for download from the FlyBase website (http://flybase.org/; Release 5) [26]. This genome is represented here with five chromosomal arms: 2L, 2R, 3L, 3R, and X (see Additional file 2), for a total of 618 markers, 114.59Mb of physical map and 249.5cM of genetic map. This dataset is manually curated and is already clean from outliers. Therefore, the cleaning step offered within BREC was skipped.

*Tomato genome* *S. lycopersicum* Domesticated tomato with 12 chromosomes has a genome size of approximately 900Mb. Based on the latest physical and genetic maps reported by the Tomato Genome Consortium [28], we present both maps content (markers number, markers density, physical map length, and genetic map length) for each chromosome in Additional file 15. For a total of 1957 markers, 752.47Mb of physical map and 1434.49cM of genetic map along the whole genome.

#### Simulated data for quality control testing

We call *data scenarios*, the layout in which the data markers are arranged along the physical map. For experimentally testing the limits of BREC, various *data scenarios* have been specifically designed based on *D. melanogaster* chromosomal arms (see Additional file 9).

In an attempt to investigate how the markers' density varies within and between the five chromosomal arms of *D. melanogaster* Release 5 genome, the density has been analyzed in two ways: locally (with 1Mb-bins) and globally (on the whole chromosome). Additional file 4 shows the results of this investigation, where each little box indicates how many markers are present within the corresponding region of size 1Mb on the physical map. The mean value represents the global density. It is also shown in Additional file 2 where the values are slightly different. This is due to computing the markers' density in two different ways with respect to the analysis. Additional file 2, presenting the genomic features of the validation dataset, shows markers density in Column 3, which is simply the result of the division of markers number (in column 2) by the physical map length (in Column 4). For example, in the case of chromosomal arm X, this gives $165/21.22 = 7.78 markers/Mb$. On the other hand, Additional file 4, aimed for analyzing the variation of local markers density, displays the mean of of all 1Mb-bins densities, which is calculated as the sum of local densities divided by the number of bins, and this gives $165/22 = 7.5 markers/Mb$.

The exact same analysis has been conducted on the tomato genome *S. lycopersicum* where the only difference lies in using 5-Mb instead of 1-Mb bins, due to the larger size of its chromosomes (see Additional file 5).

#### Validation metrics

The measure we used to evaluate the resolution of BREC's HCB is called *shift* hereafter. It is defined as the difference between the observed heterochromatin boundary ($observed\_HCB$) and the expected one ($expected\_HCB$) in terms of physical distance (in Mb)(see Equation 2).

2 $$\begin{aligned} shift = | observed\_HCB - expected\_HCB | \end{aligned}$$

The *shift* value is computed for each heterochromatin boundary independently. Therefore, we observe only two boundaries on a telocentric chromosome (one centromeric and one telomeric). In comparison, we observe four boundaries in the case of an atelocentric chromosome (two centromeric giving the centromeric region and two telomeric giving each of the two telomeric regions).

The *shift* measure was introduced not only to validate BREC's results with the reference equivalents but also to empirically calibrate the DQC module, where we are mostly interested in the variation of its value as per variations of the quality of input data.

#### Implementation and Analysis

The entire BREC project was developed using the R programming language (version 3.6.3/2020-02-29) and the RStudio environment (version 1.2.5033).

The graphical user interface is build using the shiny and shinydashboard packages. The web-based interactive plots are generated by the plotly package. Data simulations, result analysis, reproducible reports, and data visualizations are implemented using a large set of packages such as tidyverse, dplyr, R markdown, Sweave and knitr among others. The complete list of software resources used is available on the online version of the BREC package accessible at https://github.com/GenomeStructureOrganization.

From inside an R environment, the BREC package can be downloaded and installed using the command in the code chunk in Additional file 19. In case of installation issues, further documentation is available online on the ReadMe page of the GitHub repository. If all runs correctly, the BREC shiny application will be launched on your default internet browser (see Shiny interface screenshots in Additional file 14).

All BREC experiments have been carried out using a personal computer with the following specs:

- Processor: Intel$^{\textregistered}$ $\hbox {Core}^{\mathrm{TM}}$ i7-7820HQ CPU @ 2.90GHz x 8
- Memory: 32Mo
- Hard disc: 512Go SSD
- Graphics: NV117 / Mesa Intel$^{\textregistered}$ HD Graphics 630 (KBL GT2)
- Operating system: 64-bit Ubuntu 20.04 LTS

### Description of main components of the Shiny app

#### Build-in dataset

Users can either run BREC on a dataset of 44 genomes, mainly imported from [41], enriched with two mosquito genomes from [42] and updated with *D. melanogaster* Release 6 from FlyBase [26] (see Additional files 20 and 21), already available within the package, or, load new genomes data according to their own interest.

User-specific genomic data should be provided as inputs within at least a 3-column CSV file format, including for each marker: chromosome identifier, genetic distance, and physical distance, respectively. On the other hand, outputs from BREC running results are represented via interactive plots.

#### GUI input options

The BREC shiny interface provides the user with a set of options to select as parameters for a given dataset (see Fig. 3a). These options are mainly necessary in case the user works on his/her own dataset and this way the appropriate parameters would be available to choose from. First, a tab to specify the running mode (one chromosome). Then, a radio button group to choose the dataset source (existing within BREC or importing new dataset). For the existing datasets case, there is a drop-down scrolling list to select one of the available genomes (over 40 options), a second one for the corresponding physical map unit (Mb or pb) and a third one for the chromosome ID (available based on the dataset and not the genome biologically speaking). While for the import new dataset case, three more objects are added (see Fig. 3b); a fileInput to select csv data file, a textInput to enter the genome name (optional), and a drop-down scrolling list to select the data separator (comma , semicolon or tab character -set as the default-). As for the Loess regression model, the span parameter is required. It represents the percentage of how many markers to include in the local smoothing process. There is a numericInput object set by default at value 15% with an indication about the range of the span values allowed (min = 5%, max = 100%, step = 5%). The user should keep in mind that the span value actually goes from zero to one, yet, in a matter of simplification, BREC handles the conversion on its own. Thus, for example, a value of zero basically means that no markers are used for the local smoothing process by Loess, and so, it will induce a running error. Lastly, there is a checkbox to apply data cleaning if checked. Otherwise, the cleaning step will be skipped. This options could save the user some running time if s/he already have a priori knowledge that a specific genome's dataset has already been manually curated). The user is then all set to hit the Run button. BREC will start processing the chromosome of interest by identifying its type (telocentric or atelocentric). Since this step is quite difficult to automatically get the correct result, the user might be invited to interfere via a popup alert asking for a chromosome type confirmation (see Fig. 3b). As shown in Additional file 14a, all available genomes could be accessed from the left-hand panel (in dark grey) and specifically on the tab " Genomic data " where two pages are available: " Download data files " which provides a data table corresponding to the selected genome on a scrolling list along with download buttons, and " Dataset details " displaying a more global overview of the whole build-in aata repository (see Additional file 14b). To give a glance at the GUI outputs, Fig. 3c shows BREC results displayed within an interactive plot where the user will have the an interesting experience by hovering over the different plot lines and points, visualising markers labels, zooming in and out, saving a snapshot as a PNG image file, and many more available options thanks to the plotly package.

## Supplementary Information

**Additional file 1**. BREC workflow steps applied on chromosomal arm 2L of *D. melanogaster* Release 5.

**Additional file 2**. Genomic features and BREC running time for the *D. melanogaster* Release 5 genome.

**Additional file 3**. Plots representing results of BREC and reference HCB on the *D. melanogaster* genome.

**Additional file 4**. Variations of markers local density per 1-Mb bins along *D. melanogaster* Release 5 chromosomal arms.

**Additional file 5**. Variations of markers local density per 5-Mb bins along the tomato genome *S. lycopersicum* 12 chromosomes.

**Additional file 6**. Results of BREC and reference HCB on the genome of *S. lycopersicum*.

**Additional file 7**. Plots representing results of BREC and reference HCB on the *S. lycopersicum* genome.

**Additional file 8**. The impact of decreasing markers density on the resolution of BREC's HCB expressed by the shift value..

**Additional file 9**. Distribution simulations.

**Additional file 10**. A schematic description of the chromosome type identification process implemented within BREC.

**Additional file 11**. Low density simulations.

**Additional file 12**. Comparison of regression models for recombination rate estimates along the five chromosomes (X, 2L, 2R, 3L, 3R) of *D. melanogaster* Release 5.

**Additional file 13**. BREC results on different species: from top to bottom are *M. musculus* (house mouse) chromosome 4, *C. elegans* (roundworm) chromosome 3, *D. rereo*(zebrafish) chromosome 1, respectively.

**Additional file 14**. Screenshots of BREC web application - Genomic data web pages.

**Additional file 15**. Genomic features and BREC running time for *S. lycopersicum*.

**Additional file 16**. BREC workflow.

**Additional file 17**. Comparing BREC with similar widely used tools.

**Additional file 18**. The data cleaning process implemented within BREC.

**Additional file 19**. Download, install and launch BREC.

**Additional file 20**. BREC's built-in dataset of genomic data.

**Addtional file 21.** This is a spreadsheet (.xlsx file). It includes accessible links to the genetic and physical maps for the 44 genomes mentioned in Additional file 20. Adapted from the Additional file named Table S1, published by [41].

## Data Availability

The source code of the BREC R-package, including the Shiny app, is freely available at the GitHub repository https://github.com/GenomeStructureOrganization.

## Abbreviations

BREC: heterochromatin **B**oundaries and **REC**ombination rate estimates
HCB: **H**etero**C**hromatin **B**oundaries
*D*. *melanogaster*: D*rosophila melanogaster* (Fruitfly)

## References

[1] Coop G, Przeworski M (2007). An evolutionary view of human recombination. Nat Rev Genet.

[2] Duret L, Galtier N (2009). Biased gene conversion and the evolution of mammalian genomic landscapes. Annu Rev Genom Hum Genet.

[3] Auton A, McVean G (2012). Estimating recombination rates from genetic variation in humans. Methods Mol Biol.

[4] Chan AH, Jenkins PA, Song YS (2012). Genome-wide fine-scale recombination rate variation in Drosophila melanogaster. PLoS Genet.

[5] Stapley J, Feulner PGD, Johnston SE, Santure AW, Smadja CM (2017). Variation in recombination frequency and distribution across eukaryotes: patterns and processes. Philos Trans R Soc B Biol Sci.

[6] Morata J, Tormo M, Alexiou KG, Vives C, Ramos-Onsins SE, Garcia-Mas J, Casacuberta JM (2018). The evolutionary consequences of transposon-related pericentromer expansion in melon. Genome Biol Evolut.

[7] Muller H, Gil J, Drinnenberg IA (2019). The impact of centromeres on spatial genome architecture. Trends Genet.

[8] Vanrobays E, Thomas M, Tatout C. Heterochromatin positioning and nuclear architecture. In: Annual plant reviews online, pp. 157–190. Wiley, Chichester (2017). 10.1002/9781119312994.apr0502.

[9] Lu M, He X (2019). Centromere repositioning causes inversion of meiosis and generates a reproductive barrier. Proc. Natl. Acad Sci..

[10] Schueler MG, Higgins AW, Rudd MK, Gustashaw K, Willard HF (2001). Genomic and genetic definition of a functional human centromere. Science.

[11] Weinstock GM, Robinson GE, Gibbs RA, Worley KC, Evans JD,Maleszka R, Robertson HM, Weaver DB, Beye M, Bork P, Elsik CG, Hartfelder K, Hunt GJ,Zdobnov EM, Amdam GV, Bitondi MMG, Collins AM, Cristino AS, Lattorff HMG, Lobo CH,Moritz RFA, Nunes FMF, Page RE, Simões ZLP, Wheeler D, Carninci P, Fukuda S,Hayashizaki Y, Kai C, Kawai J, Sakazume N, Sasaki D, Tagami M, Albert S, Baggerman G,Beggs KT, Bloch G, Cazzamali G, Cohen M, Drapeau MD, Eisenhardt D, Emore C, Ewing MA,Fahrbach SE, Forêt S, Grimmelikhuijzen CJP, Hauser F, Hummon, AB, Huybrechts J,Jones AK, Kadowaki T, Kaplan N, Kucharski R, Leboulle G, Linial M, Littleton JT,Mercer AR, Richmond TA, Rodriguez-Zas SL, Rubin EB, Sattelle DB, Schlipalius D,Schoofs L, Shemesh Y, Sweedler JV, Velarde R, Verleyen P, Vierstraete E, Williamson MR,Ament SA, Brown SJ, Corona M, Dearden PK, Dunn WA, Elekonich MM, Fujiyuki T, Gattermeier I,Gempe T, Hasselmann M, Kadowaki T, Kage E, Kamikouchi A, Kubo T, Kucharski R,Kunieda T, Lorenzen M, Milshina NV, Morioka M, Ohashi K, Overbeek R, Ross CA,Schioett M, Shippy T, Takeuchi H, Toth AL, Willis JH, Wilson MJ, Gordon KHJ,Letunic I, Hackett K, Peterson J, Felsenfeld A, Guyer M, Solignac M, Agarwala R,Cornuet JM, Monnerot M, Mougel F, Reese JT, Schlipalius D, Vautrin D, Gillespie JJ,Cannone JJ, Gutell RR, Johnston JS, Eisen MB, Iyer VN, Iyer V, Kosarev P, Mackey AJ,Solovyev V, Souvorov A, Aronstein KA, Bilikova K, Chen YP, Clark AG, Decanini LI,Gelbart WM, Hetru C, Hultmark D, Imler JL, Jiang H, Kanost M, Kimura K, Lazzaro BP,Lopez DL, Simuth J, Thompson GJ, Zou Z, De Jong P, Sodergren E, Csurös M,Milosavljevic A, Osoegawa K, Richards S, Shu CL, Duret L, Elhaik E, Graur D,Anzola JM, Campbell KS, Childs KL, Collinge D, Crosby MA, Dickens CM, Grametes LS,Grozinger CM, Jones PL, Jorda M, Ling X, Matthews BB, Miller J, Mizzen C,Peinado MA, Reid JG, Russo SM, Schroeder AJ, St Pierre SE, Wang Y, Zhou P, Jiang H,Kitts P, Ruef B, Venkatraman A, Zhang L, Aquino-Perez G, Whitfield CW,Behura SK, Berlocher SH, Sheppard WS, Smith DR, Suarez AV, Tsutsui ND, Wei X,Wheeler D, Havlak P, Li B, Liu Y, Jovilet A, Lee S, Nazareth LV, Pu LL, Thorn R,Stolc V, Newman T, Samanta M, Tongprasit WA, Claudianos C, Berenbaum MR, Biswas S,De Graaf DC, Feyereisen R, Johnson RM, Oakeshott JG, Ranson H, Schuler MA, Muzny D,Chacko J, Davis C, Dinh H, Gill R, Hernandez J, Hines S, Hume J, Jackson LR,Kovar C, Lewis L, Miner G, Morgan M, Nguyen N, Okwuonu G, Paul H, Santibanez J,Savery G, Svatek A, Villasana D, Wright R.Insights into social insects from the genome of the honeybee Apismellifera. Nature 2006;**443**(7114), 931–949.10.1038/nature05260. arXiv:NIHMS150003.

[12] Silva-Junior OB, Grattapaglia D (2015). Genome-wide patterns of recombination, linkage disequilibrium and nucleotide diversity from pooled resequencing and single nucleotide polymorphism genotyping unlock the evolutionary history of Eucalyptus grandis. New Phytol..

[13] Robert L, Nussbaum MDFF, McInnes RR, Willard HF. Thompson and Thompson genetics in medicine. Saunders W.B. Elsevier Health Sciences (2015).

[14] Shen C, Li X, Zhang R, Lin Z (2017). Genome-wide recombination rate variation in a recombination map of cotton. PLoS ONE.

[15] Gui S, Peng J, Wang X, Wu Z, Cao R, Salse J, Zhang H, Zhu Z, Xia Q, Quan Z, Shu L, Ke W, Ding Y (2018). Improving Nelumbo nucifera genome assemblies using high-resolution genetic maps and BioNano genome mapping reveals ancient chromosome rearrangements. Plant J.

[16] Rowan BA, Heavens D, Feuerborn TR, Tock AJ, Henderson IR, Weigel D (2019). An ultra high-density arabidopsis thaliana crossover map that refines the influences of structural variation and epigenetic features. Genetics.

[17] Peñalba JV, Wolf JBW (2020). From molecules to populations: appreciating and estimating recombination rate variation. Nat Res.

[18] Stumpf MPH, McVean GAT (2003). Estimating recombination rates from population-genetic data. Nat. Rev. Genet..

[19] Jeffreys AJ (2000). High resolution analysis of haplotype diversity and meiotic crossover in the human TAP2 recombination hotspot. Hum Mol Genet.

[20] Chakravarti A (1991). A graphical representation of genetic and physical maps: the Marey map. Genomics.

[21] Rezvoy C, Charif D, Guéguen L, Marais GAB (2007). MareyMap: an R-based tool with graphical interface for estimating recombination rates. Bioinformatics.

[22] Siberchicot A, Bessy A, Guéguen L, Marais GAB (2017). MareyMap online: a user-friendly web application and database service for estimating recombination rates using physical and genetic maps. Genome Biol. Evol..

[23] Fiston-Lavier AS, Singh ND, Lipatov M, Petrov DA (2010). Drosophila melanogaster recombination rate calculator. Gene.

[24] Termolino P, Cremona G, Consiglio MF, Conicella C (2016). Insights into epigenetic landscape of recombination-free regions. Chromosoma.

[25] Langley CH, Stevens K, Cardeno C, Lee YCG, Schrider DR, Pool JE, Langley SA, Suarez C, Corbett-Detig RB, Kolaczkowski B, Fang S, Nista PM, Holloway AK, Kern AD, Dewey CN, Song YS, Hahn MW, Begun DJ (2012). Genomic variation in natural populations of Drosophila melanogaster. Genetics.

[26] Thurmond J, Goodman JL, Strelets VB, Attrill H, Gramates LS, Marygold SJ, Matthews BB, Millburn G, Antonazzo G, Trovisco V, Kaufman TC, Calvi BR, Perrimon N, Gelbart SR, Agapite J, Broll K, Crosby L, Dos Santos G, Emmert D, Falls K, Jenkins V, Sutherland C, Tabone C, Zhou P, Zytkovicz M, Brown N, Garapati P, Holmes A, Larkin A, Pilgrim C, Urbano P, Czoch B, Cripps R, Baker P (2019). FlyBase 2.0: the next generation. Nucl Acids Res.

[27] Comeron JM, Ratnappan R, Bailin S (2012). The many landscapes of recombination in *Drosophila melanogaster*. PLoS Genet.

[28] Sato S (2012). The tomato genome sequence provides insights into fleshy fruit evolution. Nature.

[29] Riddle NC, Minoda A, Kharchenko PV, Alekseyenko AA, Schwartz YB, Tolstorukov MY, Gorchakov AA, Jaffe JD, Kennedy C, Linder-Basso D, Peach SE, Shanower G, Zheng H, Kuroda MI, Pirrotta V, Park PJ, Elgin SCR, Karpen GH (2011). Plasticity in patterns of histone modifications and chromosomal proteins in *Drosophila heterochromatin*. Genome Res.

[30] Lenormand T, Engelstädter J, Johnston SE, Wijnker E, Haag CR (2016). Evolutionary mysteries in meiosis. R Soc Lond.

[31] Agresti A. An introduction to categorical data analysis, pp. 1–356. Wiley, Hoboken (2007). 10.1002/0470114754.

[32] Levan A, Fredga K, Sandberg AA (1964). Nomenclature for centromeric position on chromosomes. Hereditas.

[33] Cleveland WS, Devlin SJ (1988). Locally weighted regression: an approach to regression analysis by local fitting. J Am Stat Assoc.

[34] Cleveland WS, Loader C. Smoothing by local regression: principles and methods. In: Härdle, W., Schimek, M.G. (eds.) Statistical theory and computational aspects of smoothing, pp. 10–49. Physica-Verlag HD, Heidelberg (1996). 10.1007/978-3-642-48425-4_2.

[35] Zhang D (2017). A coefficient of determination for generalized linear models. Am Stat.

[36] Hoskins RA, Carlson JW, Kennedy C, Acevedo D, Evans-Holm M, Frise E, Wan KH, Park S, Mendez-Lago M, Rossi F, Villasante A, Dimitri P, Karpen GH, Celniker SE (2007). Sequence finishing and mapping of Drosophila melanogaster heterochromatin. Science.

[37] Hoskins RA, Carlson JW, Wan KH, Park S, Mendez I, Galle SE, Booth BW, Pfeiffer BD, George RA, Svirskas R, Krzywinski M, Schein J, Accardo MC, Damia E, Messina G, Méndez-Lago M, De Pablos B, Demakova OV, Andreyeva EN, Boldyreva LV, Marra M, Carvalho AB, Dimitri P, Villasante A, Zhimulev IF, Rubin GM, Karpen GH, Celniker SE (2015). The release 6 reference sequence of the *Drosophila melanogaster* genome. Genome Res.

[38] Cox A, Ackert-Bicknell CL, Dumont BL, Ding Y, Bell JT, Brockmann GA, Wergedal JE, Bult C, Paigen B, Flint J, Tsaih S-W, Churchill GA, Broman KW (2009). A new standard genetic map for the laboratory mouse. Genetics.

[39] Hillier LDW, Marth GT, Quinlan AR, Dooling D, Fewell G, Barnett D, Fox P, Glasscock JI, Hickenbotham M, Huang W, Magrini VJ, Richt RJ, Sander SN, Stewart DA, Stromberg M, Tsung EF, Wylie T, Schedl T, Wilson RK, Mardis ER. Whole-genome sequencing and variant discovery in *C. elegans*. Nat Methods. **5**(2), 183–188 (2008). 10.1038/nmeth.1179. 10.1038/nmeth.1179 18204455

[40] Freeman JL, Adeniyi A, Banerjee R, Dallaire S, Maguire SF, Chi J, Ng B, Zepeda C, Scott CE, Humphray S, Rogers J, Zhou Y, Zon LI, Carter NP, Yang F, Lee C (2007). Definition of the zebrafish genome using flow cytometry and cytogenetic mapping. BMC Genom.

[41] Corbett-Detig RB, Hartl DL, Sackton TB (2015). Natural selection constrains neutral diversity across a wide range of species. PLoS Biol.

[42] Dudchenko O, Batra SS, Omer AD, Nyquist SK, Hoeger M, Durand NC, Shamim MS, Machol I, Lander ES, Aiden AP, Aiden EL (2017). De novo assembly of the aedes aegypti genome using hi-c yields chromosome-length scaffolds. Science.

