# Supplementary material for: BREC: an R package/Shiny app for automatically identifying heterochromatin boundaries and estimating local recombination rates along chromosomes
Source: BMC Bioinformatics. 2021 Aug 6;22(Suppl 6):396. doi: 10.1186/s12859-021-04233-1 (PMC8349096; doi:10.1186/s12859-021-04233-1)

Figure S1: **BREC workflow steps applied on chromosomal arm 2L of *D. melanogaster* Release 5.** For each one of the five plots, the x and both y axes are the same. The x-axis represents physical distances (Mb). The left y-axis represents genetic distances (cM) shared between markers (blue data points) and the regression model (orange line). The right y-axis represents recombination rates (cM/Mb) for local estimates (green line). For simplification and less redundancy purposes, in steps 1 and 2, both y axes are written only once to be complementary for both plots: the left as well as the right one. R^2 values, varying between zero and one, are following $R^2 - forward$ (red line) and $R^2 - backwards$ (purple line). Left telomere and Right centromere (resp. black and purple dashed lines) indicate HCB for the corresponding identified heterochromatin region.

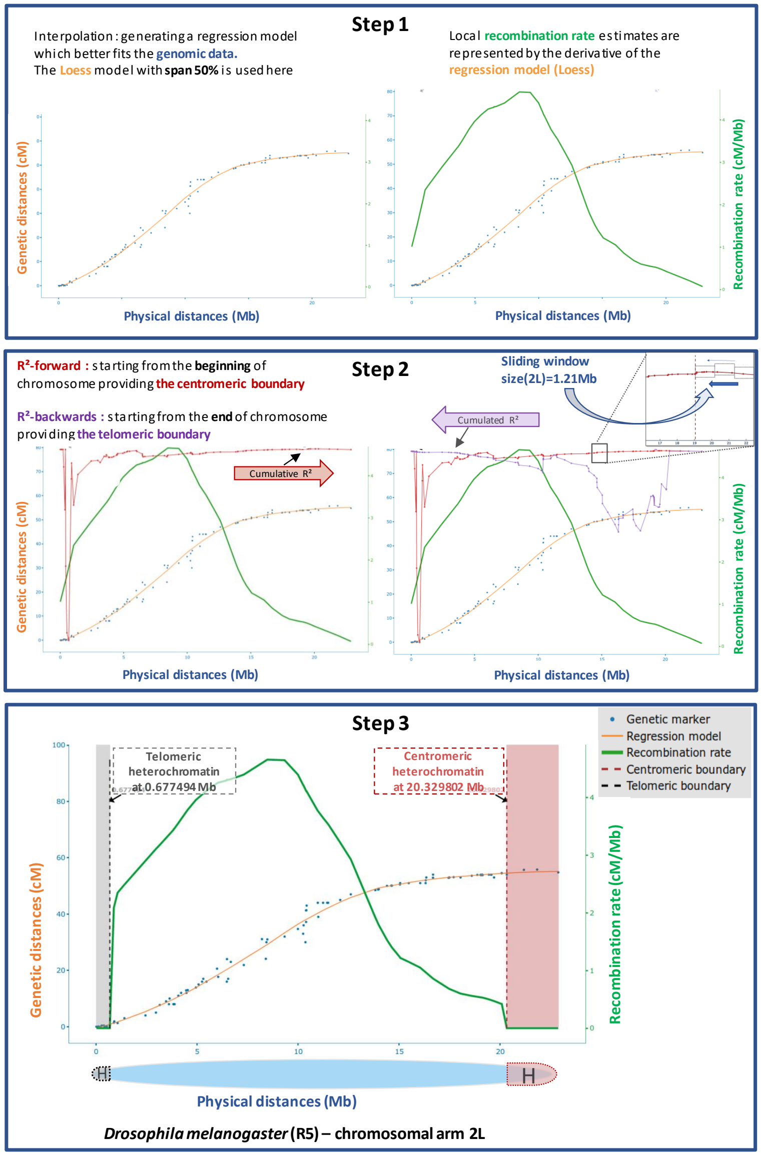

Supplement: Supplementary file 1 — Additional file 1. BREC workflow steps applied on chromosomal arm 2L of D. melanogaster Release 5. [file 12859_2021_4233_MOESM1_ESM.pdf]
