# Supplementary material for: BREC: an R package/Shiny app for automatically identifying heterochromatin boundaries and estimating local recombination rates along chromosomes
Source: BMC Bioinformatics. 2021 Aug 6;22(Suppl 6):396. doi: 10.1186/s12859-021-04233-1 (PMC8349096; doi:10.1186/s12859-021-04233-1)
Supplement: Supplementary file 2 — Additional file 2. Genomic features and BREC running time for the D. melanogaster Release 5 genome. [file 12859_2021_4233_MOESM2_ESM.pdf]

Table S1: **Genomic features and BREC running time for the *D. melanogaster* Release 5 genome**. The first five columns represent chromosomal arms. Rows represent the genome features as follows: (1) the names of chromosomal arms X, 2L, 2R, 3L, and 3R; (2) the markers number included in the study; (3) the markers density (in markers/Mb); (4) the physical map length (in Mb); (5) the genetic map length (in cM); and (6) the elapsed time when running BREC (in seconds). The last column summarises the same features for the whole genome.

**Chromosomal arms**	X	2L	2R	3L	3R	Genome
**Markers number**	165	110	101	82	160	618
**Markers density (marker/Mb)**	7.78	4.81	4.78	3.56	5.80	5.39
**Physical map length (Mb)**	21.22	22.88	21.12	21.81	27.57	114.59
**Genetic map length (cM)**	65.8	54.8	52.5	45.9	57.5	276.5
**BREC run time (sec)**	1.278	0.949	0.821	0.916	1.379	5.343
