# Supplementary material for: BREC: an R package/Shiny app for automatically identifying heterochromatin boundaries and estimating local recombination rates along chromosomes
Source: BMC Bioinformatics. 2021 Aug 6;22(Suppl 6):396. doi: 10.1186/s12859-021-04233-1 (PMC8349096; doi:10.1186/s12859-021-04233-1)

Figure S2: **Plots representing results of BREC and reference HCB on the *D. melanogaster* genome**. The results are summarized in Table 1. From top to bottom are the five chromosomal arms X, 2L, 2R, 3L, 3R, respectively. Black dots represent genetic markers in ascendant order according to their physical position (in Mb). Vertical lines represent HCB for BREC centromeres (in red dashed line), for BREC telomeres (in grey dashed line) and for the reference (in solid blue line). The heterochromatin regions identified by BREC are highlighted for the centromere (in red) and the telomere (in grey).For each chromosomal arm, two shift values of centromeric and telomeric boundaries are shown under the chromosome identifier.

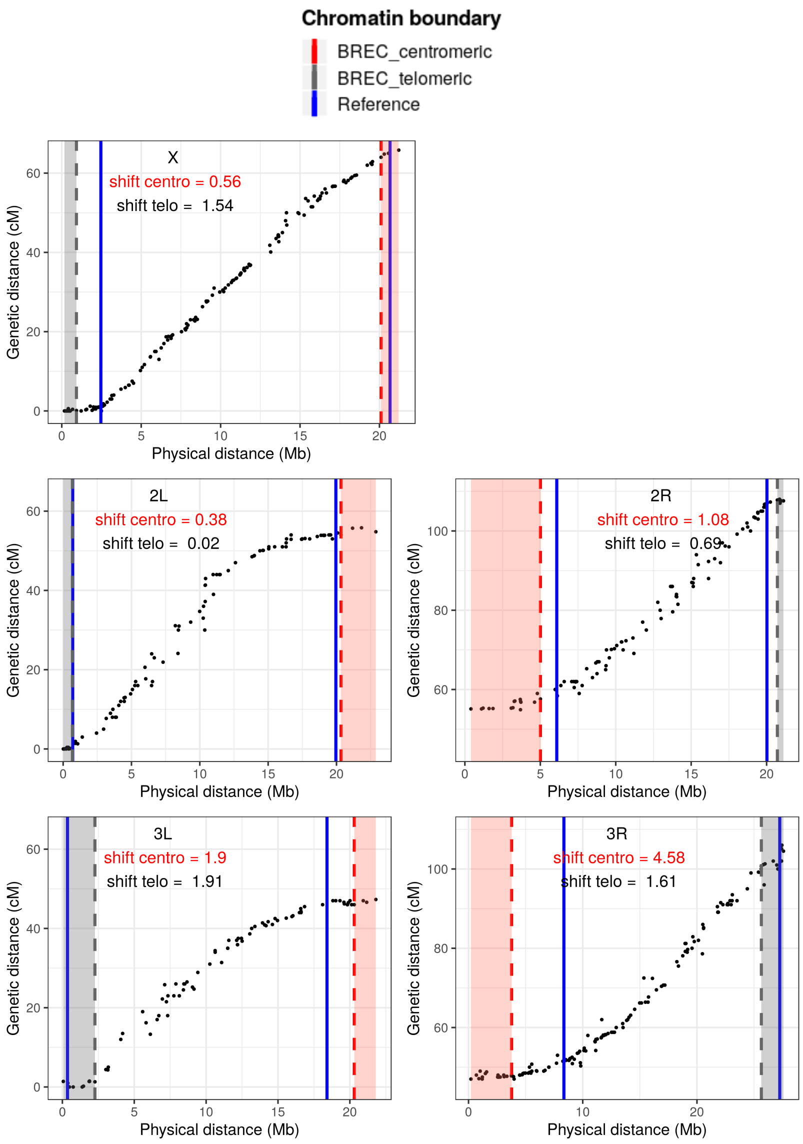

Supplement: Supplementary file 3 — Additional file 3. Plots representing results of BREC and reference HCB on the D. melanogaster genome. [file 12859_2021_4233_MOESM3_ESM.pdf]
