# Supplementary material for: BREC: an R package/Shiny app for automatically identifying heterochromatin boundaries and estimating local recombination rates along chromosomes
Source: BMC Bioinformatics. 2021 Aug 6;22(Suppl 6):396. doi: 10.1186/s12859-021-04233-1 (PMC8349096; doi:10.1186/s12859-021-04233-1)

Figure S3: **Variations of markers local density per 1-Mb bins along *D. melanogaster* Release 5 chromosomal arms.** The red dashed line indicates the mean and represents the global density. Each bin indicates the number of markers it contains. Local density values are represented within the little boxes.

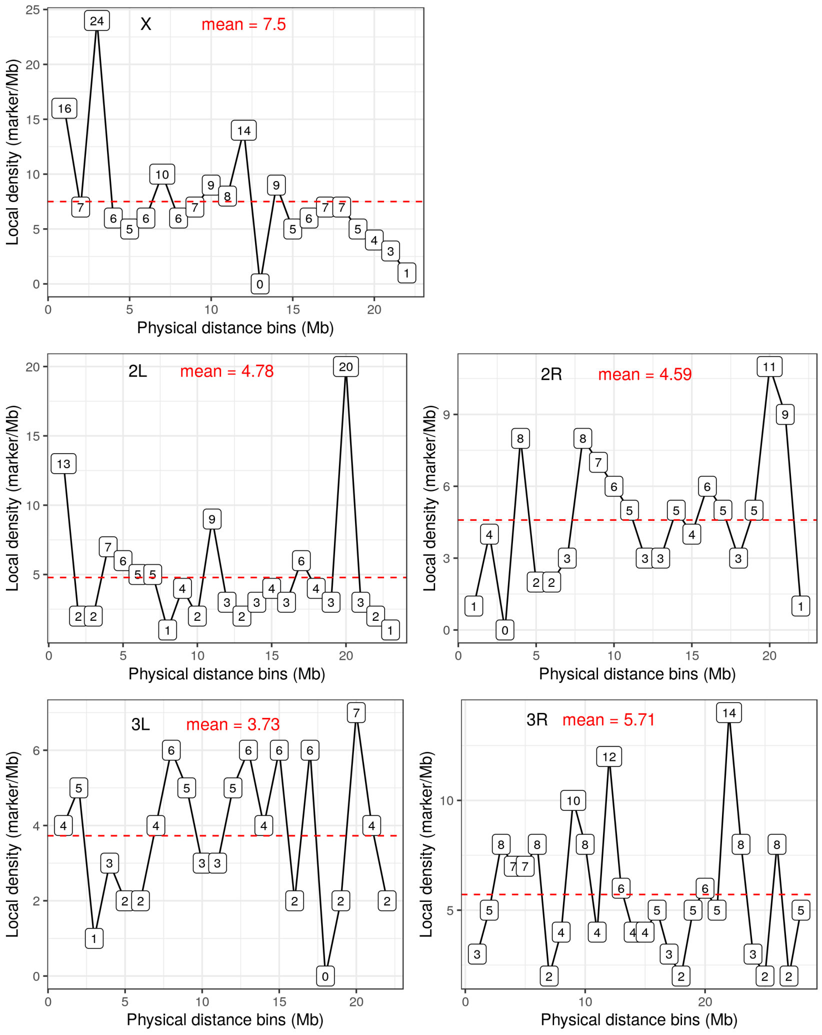

Supplement: Supplementary file 4 — Additional file 4. Variations of markers local density per 1-Mb bins along D. melanogaster Release 5 chromosomal arms. [file 12859_2021_4233_MOESM4_ESM.pdf]
