# Supplementary material for: BREC: an R package/Shiny app for automatically identifying heterochromatin boundaries and estimating local recombination rates along chromosomes
Source: BMC Bioinformatics. 2021 Aug 6;22(Suppl 6):396. doi: 10.1186/s12859-021-04233-1 (PMC8349096; doi:10.1186/s12859-021-04233-1)
Supplement: Supplementary file 6 — Additional file 6. Results of BREC and reference HCB on the genome of S. lycopersicum. [file 12859_2021_4233_MOESM6_ESM.pdf]

Table S2: **Results of BREC and reference HCB on the genome of *S. lycopersicum*.** The shift is the absolute value of the distance between the BREC and the reference physical heterochromatin boundary. The first twelve rows represent all chromosomes. Grouped columns present reference, BREC and shift values for the left centromeric boundaries (Columns 2-4), and for the right centromeric boundaries (Columns 4-6). All values are expressed in Megabase (Mb). The red asterisk indicates the largest shift value reported on centromeric and telomeric boundaries separately (see corresponding Additional file 7). The last four rows represent some general statistics on the shift value. From top to bottom, they are minimum, maximum, mean, and median respectively. See details on the shift metrics in Section Materials and Methods: Validation metrics.

**Chromosome**	**Centromeric left (Mb)**			**Centromeric right (Mb)**		
	**Boundaries**		**Shift**	**Boundaries**		**Shift**
	**Reference**	**BREC**		**Reference**	**BREC**	
**1**	5.78	22.88	17.09	67.80	76.48	8.68
**2**	3.15	1.51	1.64	27.43	21.31	6.12
**3**	5.75	6.98	1.23	55.34	49.28	6.06
**4**	5.48	1.21	4.27	54.92	47.21	7.72
**5**	6.02	15.03	9.01	60.23	51.04	9.19
**6**	1.50	1.68	0.19	29.62	20.42	9.20
**7**	5.62	23.05	17.43	52.51	33.52	18.98*
**8**	5.10	22.87	17.77	51.73	43.96	7.77
**9**	4.38	32.51	28.12*	61.16	49.16	12.00
**10**	4.40	24.37	19.97	58.83	49.92	8.91
**11**	5.56	10.86	5.29	47.57	32.77	14.80
**12**	7.27	14.34	7.07	60.27	54.33	5.94
**Min. shift**	0.19			5.94		
**Max. shift**	28.12			18.98		
**Mean shift**	10.76			9.61		
**Median shift**	8.04			8.80		
