# Supplementary material for: BREC: an R package/Shiny app for automatically identifying heterochromatin boundaries and estimating local recombination rates along chromosomes
Source: BMC Bioinformatics. 2021 Aug 6;22(Suppl 6):396. doi: 10.1186/s12859-021-04233-1 (PMC8349096; doi:10.1186/s12859-021-04233-1)

Figure S5: **Plots representing results of BREC and reference HCB on the *S. lycopersicum* genome**. The results are summarized in Additional file 6. From top to bottom are the twelve chromosomes 1 to 12, respectively. Black dots represent genetic markers in ascendant order according to their physical position (in Mb). Vertical lines represent HCB for BREC centromeres (in red dashed line), and for the reference (in solid blue line). The heterochromatin regions identified by BREC are highlighted for the centromere (in red). Rug plot on the x-axis represents the markers density according to the physical map.

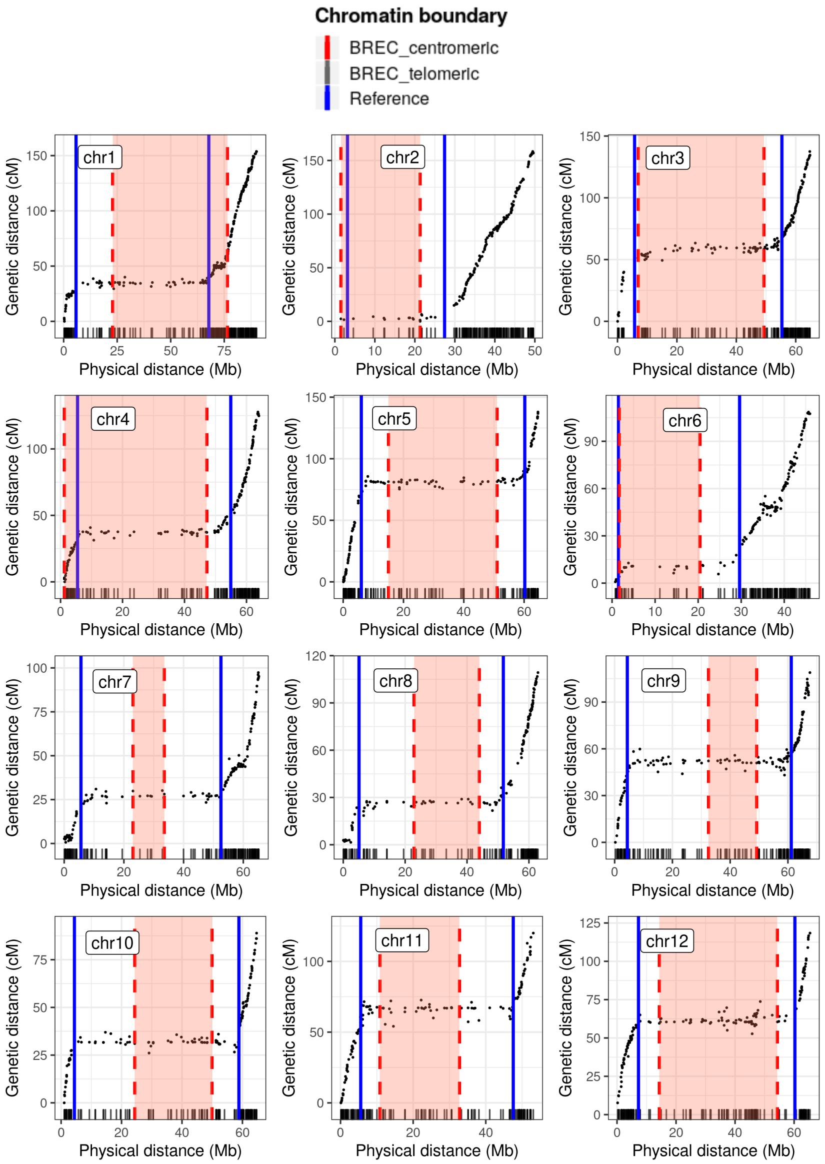

Supplement: Supplementary file 7 — Additional file 7. Plots representing results of BREC and reference HCB on the S. lycopersicum genome. [file 12859_2021_4233_MOESM7_ESM.pdf]
