# Supplementary material for: BREC: an R package/Shiny app for automatically identifying heterochromatin boundaries and estimating local recombination rates along chromosomes
Source: BMC Bioinformatics. 2021 Aug 6;22(Suppl 6):396. doi: 10.1186/s12859-021-04233-1 (PMC8349096; doi:10.1186/s12859-021-04233-1)

Figure S6: **The impact of decreasing markers density on the resolution of BREC's HCB expressed by the shift value.** Here is an overview of the variation of shift values (see Equation 2) for BREC's HCB compared to reference results for the five *D. melanogaster* chromosomal arms (X, 2L, 2R, 3L, 3R). For each arm, two HCB are shown: squares (in red) for telomeric and triangles (in light blue) for centromeric boundaries. The horizontal dashed line (in black) delimits results smaller than a shift value of 3Mb for all arms while the vertical dashed line (in black) indicates up to which fraction the 3Mb shift is conserved on each chromosomal arm's simulations. Note that the x axis is reversed, so from left to right it goes from 100% to 30% with a step of -5%at each point. The simulation process is further clarified for one fraction on the chromosomal arm 2L and is illustrated in Additional file 11.

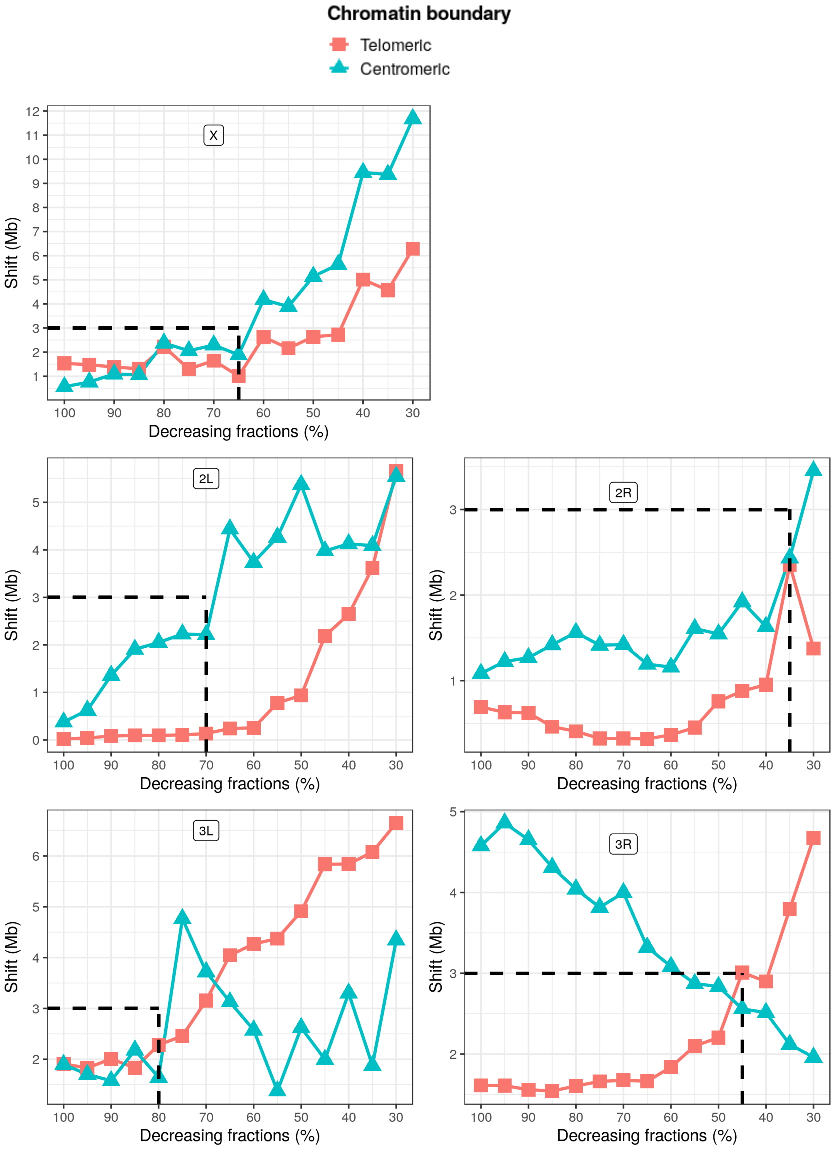

Supplement: Supplementary file 8 — Additional file 8. The impact of decreasing markers density on the resolution of BREC's HCB expressed by the shift value.. [file 12859_2021_4233_MOESM8_ESM.pdf]
