# Supplementary material for: BREC: an R package/Shiny app for automatically identifying heterochromatin boundaries and estimating local recombination rates along chromosomes
Source: BMC Bioinformatics. 2021 Aug 6;22(Suppl 6):396. doi: 10.1186/s12859-021-04233-1 (PMC8349096; doi:10.1186/s12859-021-04233-1)

Figure S7: **Distribution simulations.** BREC results on the simulated chromosomes with different scenarios of markers distribution around heterochromatin regions, as presented in the table (top) . Plots (right after) are presenting the corresponding results for each simulation scenario. On the left, (a, c, e) show the cases with the existence of centromeric gap while the ones on the right (b, d, f) show the cases with no centromeric gap. From top to bottom, cases (a) and (b) show a uniform distributions while (c) to (f) are for non uniform distributions. Cases (c) and (d) show a higher density of markers around heterochromatin regions while cases (e) and (f) show a lower density on the same regions. Black dots represent genetic markers. Vertical lines represent HCB for BREC centromeres (in red dashed line), for BREC telomeres (in grey dashed line) and for the reference (in solid blue line). The heterochromatin regions identified by BREC are highlighted for the centromere (in red) and the telomere (in grey). The rug plot, added on the x axis, shows more clearly the variation in markers density as well as the existence or not of the centromeric gap.

Simulation Data scenarios	Centromeric gap exists (lack of markers)		Markers density on heterochromatic regions
	Yes	No	
Uniform distribution	(a)	(b)	Unvariant
Non uniform distribution	(c)	(d)	High
	(e)	(f)	Low

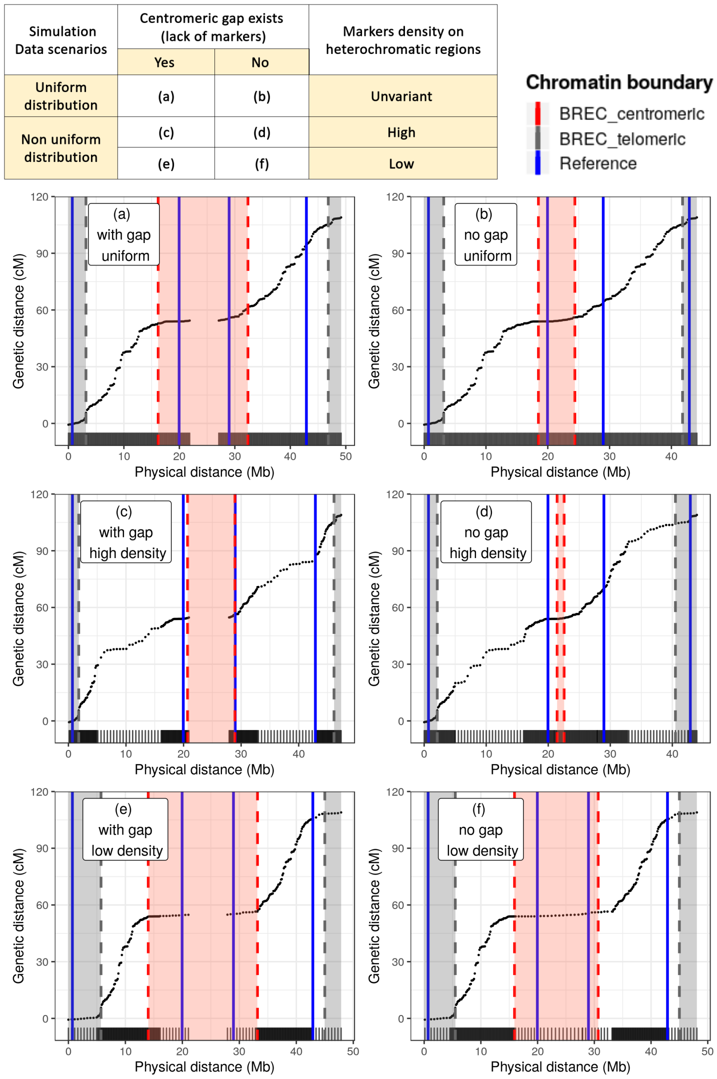

Supplement: Supplementary file 9 — Additional file 9. Distribution simulations. [file 12859_2021_4233_MOESM9_ESM.pdf]
