# Supplementary material for: BREC: an R package/Shiny app for automatically identifying heterochromatin boundaries and estimating local recombination rates along chromosomes
Source: BMC Bioinformatics. 2021 Aug 6;22(Suppl 6):396. doi: 10.1186/s12859-021-04233-1 (PMC8349096; doi:10.1186/s12859-021-04233-1)

Figure S8: **A schematic description of the chromosome type identification process implemented within BREC.** (a) Telocentric chromosome type is when the centromere (the grey colored circle) is located on one of the chromosomal arm extremities (indicated with the green upside down triangle). (b) Atelocentric chromosome type -confirmed as metacentric- is when the centromere is located approximately on the middle of the chromosome, here showed within the physical positions 40% and 60% of the chromosome's size (delimited by the red brackets and indicated with the tag "Meta"). (c) Atelocentric chromosome type -with no specification- is when the centromere is located either inside the first arm (between the beginning of the chromosome and 40% of its size), or inside the second arm (between 60% and the end, indicated with the tag "Don't know").

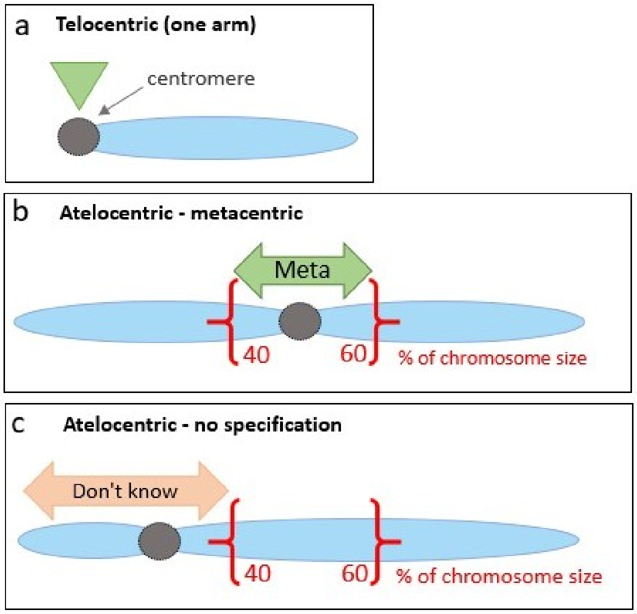

Supplement: Supplementary file 10 — Additional file 10. A schematic description of the chromosome type identification process implemented within BREC. [file 12859_2021_4233_MOESM10_ESM.pdf]
