# Supplementary material for: BREC: an R package/Shiny app for automatically identifying heterochromatin boundaries and estimating local recombination rates along chromosomes
Source: BMC Bioinformatics. 2021 Aug 6;22(Suppl 6):396. doi: 10.1186/s12859-021-04233-1 (PMC8349096; doi:10.1186/s12859-021-04233-1)

Figure S9: **Low density simulations** BREC results on the simulated telocentric chromosomes with different density scenarios. Simulating decreasing markers density going from 100% to 30% of the original chromosome 2L (of size 23Mb) of the *D. melanogaster* Release 5 genome. These simulations allow to study the impact of variable markers density on BREC results compared to reference HCB. (a) on the left is before and (b) on the right is after the cleaning step. These simulations have been conducted on each of the five chromosomes (X, 2L, 2R, 3L, 3R) 30 times where the mean shift value is reported in Additional file 8. Black dots represent genetic markers. Vertical lines represent HCB for BREC centromeres (in red dashed line), for BREC telomeres (in grey dashed line) and for the reference (in solid blue line). The heterochromatin regions identified by BREC are highlighted for the centromere (in red) and the telomere (in grey). The corresponding fraction and markers density is shown on the top left of each simulation plot.

(a) Before cleaning

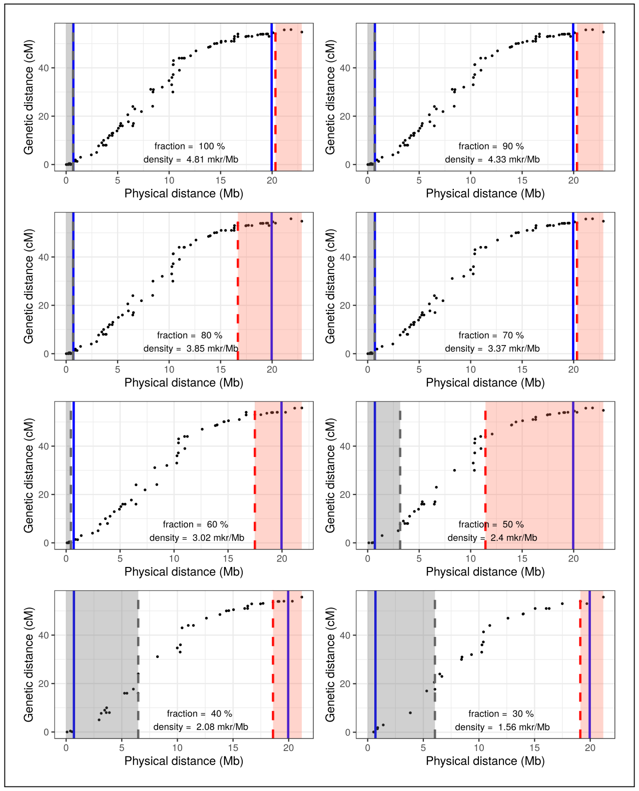

(b) After cleaning

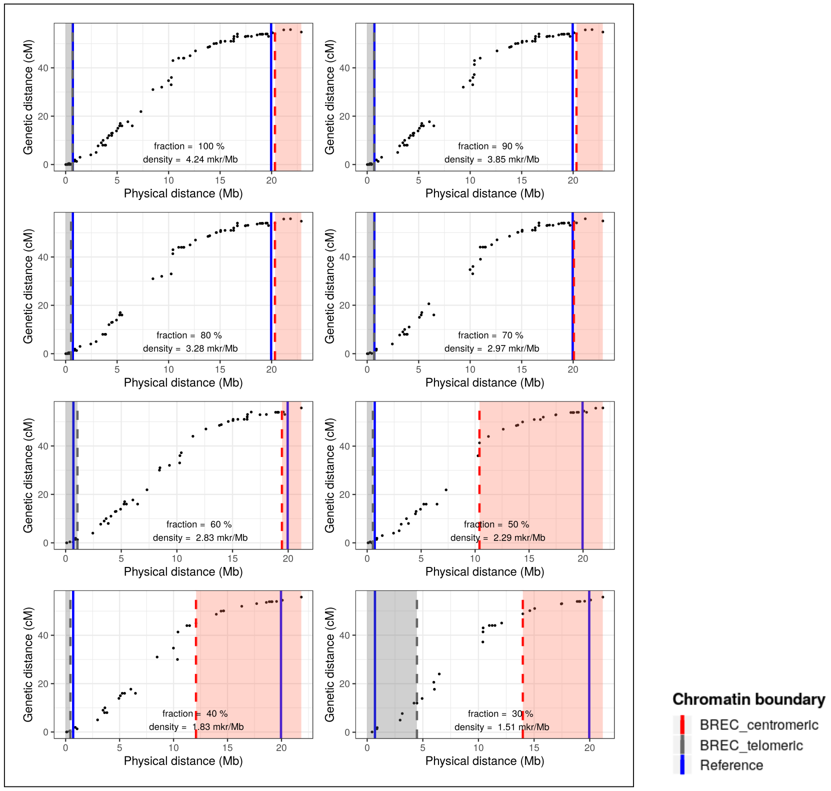

Supplement: Supplementary file 11 — Additional file 11. Low density simulations. [file 12859_2021_4233_MOESM11_ESM.pdf]
