# Supplementary material for: BREC: an R package/Shiny app for automatically identifying heterochromatin boundaries and estimating local recombination rates along chromosomes
Source: BMC Bioinformatics. 2021 Aug 6;22(Suppl 6):396. doi: 10.1186/s12859-021-04233-1 (PMC8349096; doi:10.1186/s12859-021-04233-1)

Figure S10: **Comparison of regression models for recombination rate estimates along the five chromosomes (X, 2L, 2R, 3L, 3R) of *D. melanogaster* Release 5**. Regression models used here are Loess with span values, 15%, 25%, 50%, 75% and third degree polynomial. The HCB defined by BREC remain unchanged and only local recombination rates differ according to the model used to fit the genetic and physical maps. Recombination rate is represented by the derivative of the model. In case of two or more models yielding the same recombination rate estimates on the same physical position, the overlap results in only one curve line. Here, all curves show null recombination rate value on the centromeric and telomeric regions.

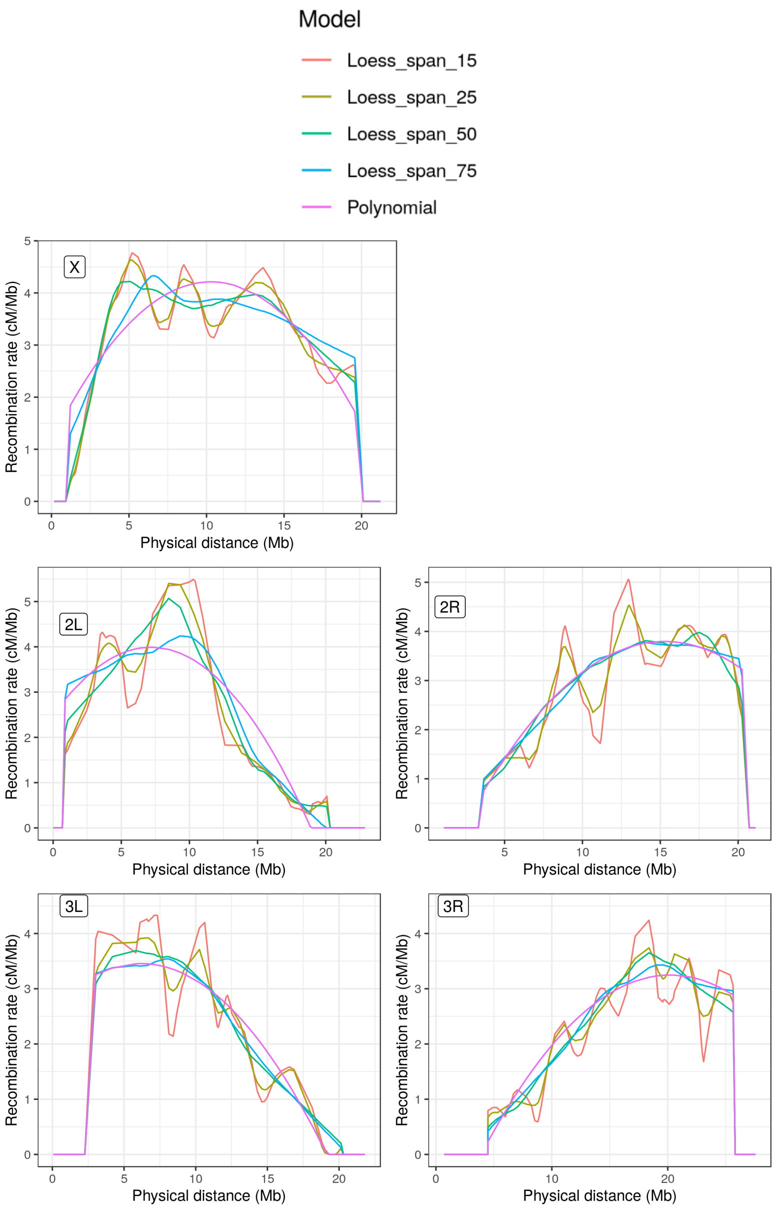

Supplement: Supplementary file 12 — Additional file 12. Comparison of regression models for recombination rate estimates along the five chromosomes (X, 2L, 2R, 3L, 3R) of D. melanogaster Release 5. [file 12859_2021_4233_MOESM12_ESM.pdf]
