# Supplementary material for: BREC: an R package/Shiny app for automatically identifying heterochromatin boundaries and estimating local recombination rates along chromosomes
Source: BMC Bioinformatics. 2021 Aug 6;22(Suppl 6):396. doi: 10.1186/s12859-021-04233-1 (PMC8349096; doi:10.1186/s12859-021-04233-1)

Figure S11: **BREC results on different species: from top to bottom are *M. musculus* (house mouse) chromosome 4, *C. elegans* (roundworm) chromosome 3, *D. rereo* (zebrafish) chromosome 1, respectively.** For each species, two plots are shown: on the left is the chromosome's genetic markers (black points), their distribution along the physical map (rug on the x-axis), and reported genomic features (label in blue). On the right is BREC results: HCB for centromeric (red highlight) and telomeric (grey highlight) regions, (RR) local recombination rate estimates (red line), and the running time of BREC's algorithms to get these results (loading data and plotting are excluded).

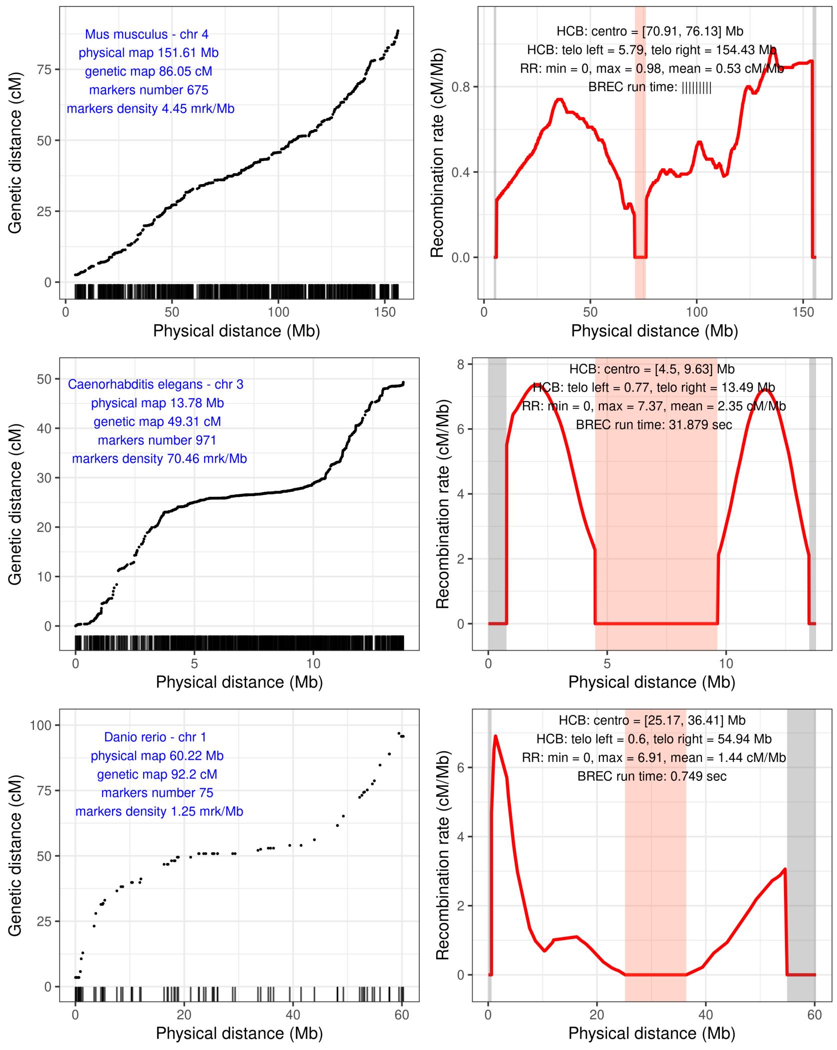

Supplement: Supplementary file 13 — Additional file 13. BREC results on different species: from top to bottom are M. musculus (house mouse) chromosome 4, C. elegans (roundworm) chromosome 3, D. rereo(zebrafish) chromosome 1, respectively. [file 12859_2021_4233_MOESM13_ESM.pdf]
