# Supplementary material for: BREC: an R package/Shiny app for automatically identifying heterochromatin boundaries and estimating local recombination rates along chromosomes
Source: BMC Bioinformatics. 2021 Aug 6;22(Suppl 6):396. doi: 10.1186/s12859-021-04233-1 (PMC8349096; doi:10.1186/s12859-021-04233-1)

Figure S12: **Screenshots of BREC web application - Genomic data web pages.**

(a) Top

Download data files page from the Genomic data section, indicated on the left dark panel, is displayed here. After selecting on the top list the *Gallus gallus* genome and clicking the "Download selected" button, a dialog box is open waiting for the user to specify the file path to save the selected data file.

(b) Bottom

Dataset details page from the Genomic data section is showing a sample of ten available genomes provided within the BREC package. The table is intentionally sorted using the forth column values with descending number of "Total markers".

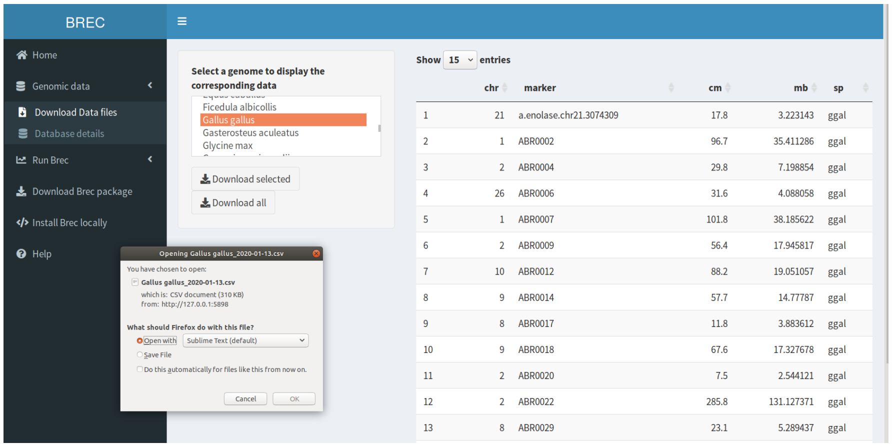

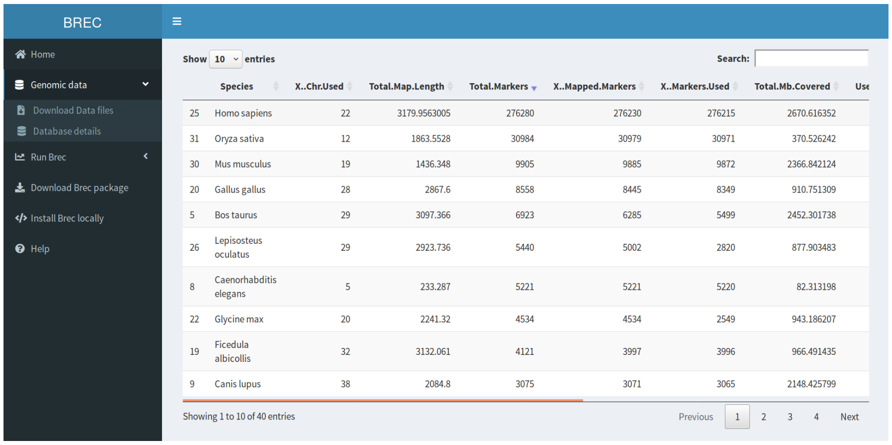

Supplement: Supplementary file 14 — Additional file 14. Screenshots of BREC web application - Genomic data web pages. [file 12859_2021_4233_MOESM14_ESM.pdf]
