# Supplementary material for: BREC: an R package/Shiny app for automatically identifying heterochromatin boundaries and estimating local recombination rates along chromosomes
Source: BMC Bioinformatics. 2021 Aug 6;22(Suppl 6):396. doi: 10.1186/s12859-021-04233-1 (PMC8349096; doi:10.1186/s12859-021-04233-1)
Supplement: Supplementary file 15 — Additional file 15. Genomic features and BREC running time for S. lycopersicum. [file 12859_2021_4233_MOESM15_ESM.pdf]

Table S3: **Genomic features and BREC running time for the *S. lycopersicum*** . The first twelve columns represent chromosomes. Rows represent the genome features as follows: (1) the identifiers of chromosomes 1 to 12; (2) the markers number included in the study; (3) the markers density (in markers/Mb); (4) the physical map length (in Mb); (5) the genetic map length (in cM); and (6) the elapsed time when running BREC (in seconds). The last column summarises the same features for the whole genome.

**Chromosome**	1	2	3	4	5	6	7	8	9	10	11	12	Genome
**Markers number**	232	176	184	160	150	151	145	144	171	148	142	154	1957
**Markers density (marker/Mb)**	2.58	3.66	2.84	2.55	2.32	3.34	2.22	2.29	2.54	2.32	2.68	2.36	2.64
**Physical map length (Mb)**	89.85	48.10	64.77	62.79	64.52	45.20	65.18	62.87	67.37	63.66	52.98	65.18	752.47
**Genetic map length (cM)**	150.72	154.58	134.52	122.64	137.91	106.63	92.48	106.63	108.90	88.92	119.99	110.72	1434.49
**BREC run time (sec)**	2.164	1.391	1.434	1.295	1.098	1.197	1.102	1.047	1.357	1.095	1.081	1.221	15.479
