# Supplementary material for: BREC: an R package/Shiny app for automatically identifying heterochromatin boundaries and estimating local recombination rates along chromosomes
Source: BMC Bioinformatics. 2021 Aug 6;22(Suppl 6):396. doi: 10.1186/s12859-021-04233-1 (PMC8349096; doi:10.1186/s12859-021-04233-1)

Figure S13: **BREC workflow.** As a more detailed version of Fig 1, this figure provides an overview of the tool design explaining how the different modules are linked together and how BREC functionalities are implemented. The left part represents the top-to-bottom diagram, starting with the required input data, how they are pre-processed (Step 0) and exploited (Main process), then, what outputs are expected to be returned and in which format. The right part of the figure, representing a zoom-in on BREC's main module (estimating recombination rates, identifying chromosome type, identifying HCB, extrapolating the recombination map and generating the interactive plot), clarifies each step following a more detailed scheme.

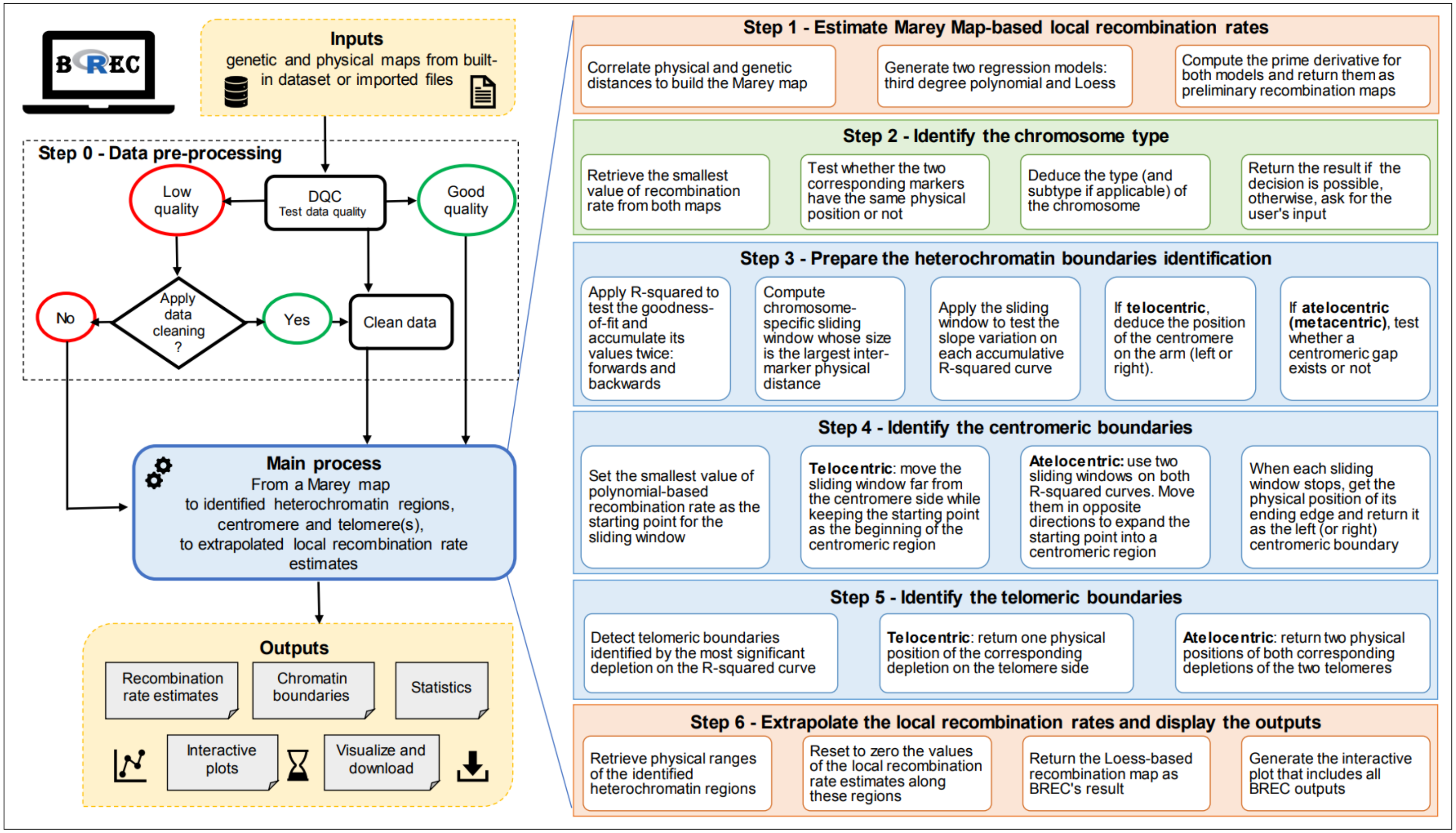

Supplement: Supplementary file 16 — Additional file 16. BREC workflow. [file 12859_2021_4233_MOESM16_ESM.pdf]
