# Supplementary material for: BREC: an R package/Shiny app for automatically identifying heterochromatin boundaries and estimating local recombination rates along chromosomes
Source: BMC Bioinformatics. 2021 Aug 6;22(Suppl 6):396. doi: 10.1186/s12859-021-04233-1 (PMC8349096; doi:10.1186/s12859-021-04233-1)
Supplement: Supplementary file 17 — Additional file 17. Comparing BREC with similar widely used tools. [file 12859_2021_4233_MOESM17_ESM.pdf]

Table S4: **Comparing BREC with similar widely used tools.** BREC's provided features and functionalities are compared along with the Recombination Rate Calculator (RRC) [23] and the MareyMapOnline [22], following a chronological order (the oldest first).

Features / Tool		RRC	MareyMap Online	BREC
**Publication year**		2010	2017	2020
**Genome-specific**		D. melanogaster	non-specific	non-specific
**Interpolation method**	**Polynomial**	yes	yes	no
	**Loess**	no	yes	yes
	**Cubic spline**	no	yes	no
**Data cleaning**		no	manual	automatic
**Data Quality Control**		no	no	yes
**Chromatin boundaries identification**		no	no	yes
**Software**	R package	no	yes	yes
	**Web-based GUI**	Perl CGI	Shiny	Shiny
