# Supplementary material for: BREC: an R package/Shiny app for automatically identifying heterochromatin boundaries and estimating local recombination rates along chromosomes
Source: BMC Bioinformatics. 2021 Aug 6;22(Suppl 6):396. doi: 10.1186/s12859-021-04233-1 (PMC8349096; doi:10.1186/s12859-021-04233-1)

Figure S14: **The data cleaning process implemented within BREC**. Inter-marker distances (*i.e.* genetic distances between each two consecutive points along the genetic map) are represented using a boxplot in order to identify outliers and give the user the option to remove them. Here is an example showing raw data of a simulated chromosome (left) with the specific markers detected as outliers (red dots circled with red dashed ovals) and the corresponding genetic distances (also in red) on the boxplot (right).

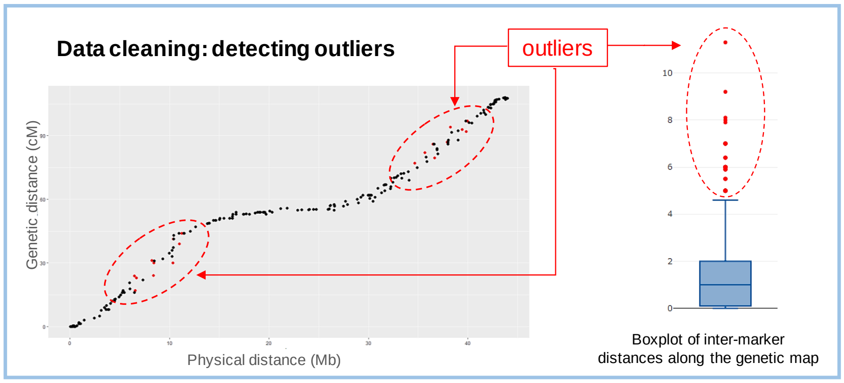

Supplement: Supplementary file 18 — Additional file 18. The data cleaning process implemented within BREC. [file 12859_2021_4233_MOESM18_ESM.pdf]
