# Supplementary material for: BREC: an R package/Shiny app for automatically identifying heterochromatin boundaries and estimating local recombination rates along chromosomes
Source: BMC Bioinformatics. 2021 Aug 6;22(Suppl 6):396. doi: 10.1186/s12859-021-04233-1 (PMC8349096; doi:10.1186/s12859-021-04233-1)
Supplement: Supplementary file 19 — Additional file 19. Download, install and launch BREC. [file 12859_2021_4233_MOESM19_ESM.pdf]

Figure S15: **Download, install and launch BREC.** Code chunk showing the R commands allowing to download, install and run the BREC shiny application. The entire R package is available with open access on the indicated GitHub repository.

```r
# Install devtools and shiny from CRAN
install.packages("devtools", "shiny")
# Load installed libraries
library(devtools, shiny)


# Download and install the BREC package from the GitHub repository
install_github("ymansour21/BREC")
# Load Brec and shiny
library(Brec)
library(shiny)
# Launch Brec graphical interface in your default internet browser
runApp("shinyApp/Brec_dashboard.R", launch.browser = TRUE)
```
