# Supplementary material for: BREC: an R package/Shiny app for automatically identifying heterochromatin boundaries and estimating local recombination rates along chromosomes
Source: BMC Bioinformatics. 2021 Aug 6;22(Suppl 6):396. doi: 10.1186/s12859-021-04233-1 (PMC8349096; doi:10.1186/s12859-021-04233-1)
Supplement: Supplementary file 20 — Additional file 20. BREC's built-in dataset of genomic data. [file 12859_2021_4233_MOESM20_ESM.pdf]

Table S5: **BREC's built-in dataset of genomic data.** The available genetic and physical maps for 44 species from [41], enriched with two recently assembled mosquito genomes: *Culex pipiens* and *Aedes aegypti* from [42], domesticated tomato *S. lycopersicum* from [28], and *D. melanogaster* Release 6 (update) from FlyBase [26] (see Additional file 21 — Table S6). The species in red bold text are the ones used in BREC experiments. Since the data collection process is still ongoing, the current version of this dataset is continuously evolving.

Species	Common Name	Taxonomy
Aedes aegypti	Yellow fever mosquito	Animal Invertebrate
Anopheles gambiae	African malaria mosquito	
Apis mellifera scutellata	Honeybee	
Bombyx mandarina	Silkworm	
Caenorhabditis briggsae	Roundworm	
**Caenorhabditis elegans**	Roundworm	
Culex pipiens	Common house mosquito	
**Drosophila melanogaster R5**	Fruit fly	
Drosophila melanogaster R6	Fruit fly	
Drosophila pseudoobscura	Fruit fly	
Heliconius melpomene melpomene	Postman butterfly	
Bos taurus	Cow	Animal Vertebrate
Canis lupus	Wolf	
Cynoglossus semilaevis	Tongue sole	
**Danio rerio**	Zebrafish	
Equus ferus przewalskii	Prewalksii's horse	
Ficedula albicollis	Collared flycatcher	
Gallus gallus	Chicken	
Gasterosteus aculeatus	Stickleback	
Homo sapiens	Human	
Lepisosteus oculatus	Spotted gar	
Macaca mulatta	Rhesus macaque	
Meleagris gallopavo	Turkey	
**Mus musculus castaneus**	House mouse	
Oryzias latipes	Medaka	
Ovis canadensis	Bighorn sheep	
Papio anubis	Olive baboon	
Sus scrofa	Wild boar	
Citrus reticulata	Mandarin Orange	Plant Woody
Gossypium raimondii	New world cotton	
Populus trichocarpa	Black cottonwood	
Prunus davidiana	David's peach	
Arabidopsis thaliana	Thale cress	Plant Herbaceous
Brachypodium distachyon	Purple false brome	
Capsella rubella	Pink Shepherd's Purse	
Citrullus lanatus lanatus	Watermellon	
Cucumis sativus var. hardwickii	Cucumber	
Glycine soja	Wild soybean	
Medicago truncatula	Barrel medic	
Oryza rufipogon	Wild rice	
Setaria italica	Foxtail millet	
Sorghum bicolor subsp. verticilliflorum	Wild Sudan grass	
**Solanum lycopersicum**	Domesticated tomato	
Zea mays ssp parviglumis	Teosinte	
